# Cetuximab co-treatment with KRAS G12C inhibitors fulzerasib and sotorasib in human KRAS G12C non-small cell lung cancer cells

**DOI:** 10.1038/s41420-026-02998-z

**Published:** 2026-03-05

**Authors:** Daniel Olmo-González, Mengxin Zhou, Nuno G. Oliveira, Fusheng Zhou, Feng Yan, Jèssica González, Kevin València-Clua, Miguel Angel Molina-Vila, Jordi Bertrán-Alamillo, Ana Giménez-Capitán, Jordi Codony-Servat, Rafael Rosell

**Affiliations:** 1 FIOR Group, Dexeus University Hospital, Barcelona, Spain; 2 Department of Biochemistry and Molecular Biomedicine, University of Barcelona, Faculty of Biology, Barcelona, Spain; 3 FIOR Group, FIGTP - Fundació Institut d’Investigació Germans Trias i Pujol, Badalona, Spain; 4 Research Institute for Medicines (iMed.ULisboa), Faculty of Pharmacy, Universidade de Lisboa, Lisboa, Portugal; 5 GenFleet Therapeutics (Shanghai) Inc., Shanghai, China; 6 Pangaea Oncology, Dexeus University Hospital, Barcelona, Spain; 7 Instituto Oncológico Dr. Rosell-IOR, Dexeus University Hospital, Barcelona, Spain

**Keywords:** Non-small-cell lung cancer, Tumour biomarkers

## Abstract

Resistance to KRAS G12C inhibitors is a major cause of poor prognosis in non-small cell lung cancer (NSCLC). Sotorasib and fulzerasib target the KRAS protein in the guanosine diphosphate (GDP)-bound inactive state. Used in monotherapy in non-small cell lung cancer (NSCLC) patients, these drugs yielded response rates of 41% and 49% and median progression-free survival of 6.3 and 9.7 months, respectively. Mounting evidence points out that feedback activation of epidermal growth factor receptor (EGFR) inhibits the hydrolysis of KRAS guanosine triphosphate (GTP)-bound active state and reactivates RAS-mitogen-activated protein kinase (MAPK) signaling, leading to drug resistance. Here, we demonstrated that cetuximab enhanced the antitumor growth effect of fulzerasib in vitro in H358 cells and prolonged the survival of mice bearing subcutaneous H358 xenografts. Moreover, we demonstrated that cotreatment suppressed the expression of ERK, AKT, as well as MRAS and YAP1. Additionally, the combination of fulzerasib with cetuximab also abolished the viability of H358 cells, being less effective for H23 and H2030 cells. This effect was accompanied by YAP1 and MRAS overexpression, especially in H2030 cells. Ultimately, we demonstrated that MIG6, a feedback negative regulator of EGFR, is suppressed in the three cell lines starting at the 24-h time point with fulzerasib or sotorasib. Concurrently, ASS1, initially low in all three cell lines, was upregulated following therapy with fulzerasib or sotorasib, with or without cetuximab. These alterations were associated with increased fumarate levels in all three cell lines examined. Notably, the cotreatment response of H358 cells mirrors clinical trials with KRAS G12C colorectal cancer and NSCLC patients, where the combination of cetuximab with KRAS G12C inhibitors resulted in increased response and progression-free survival. Our findings provide insight into further tailoring EGFR inhibitor cotreatment in KRAS G12C NSCLC patients.

## Introduction

RAS mutations were detected in 19 of 66 non-small cell lung cancer (NSCLC) cell lines (29%); eleven occurred at codon 12 of Kirsten rat sarcoma viral oncogene homologue (KRAS), and six consisted of GGT to TGT mutations [1]. For patients who received palliative treatment, detection of KRAS mutations at codon 12 is associated with shortened survival (*P* = 0.01) [2]. Among 295 patients with KRAS G12C-mutant advanced NSCLC, the median overall survival and progression-free survival with docetaxel regimens as second-line therapy were 6.0 and 3.4 months, respectively [3]. Sotorasib (AMG-510) inhibitor, covalently binds to KRAS G12C only in the inactive GDP-bound conformation, and the objective response to sotorasib was observed in 46 of 124 patients previously treated with platinum-based chemotherapy and anti-PD-1(or PD-L1) antibodies (37.1%), including 4 (3.2%) who attained a complete response. The median progression-free survival was 6.8 months, while the median overall survival was 12.5 months (CodeBreak 100 clinical trial) [4]. When sotorasib was compared with docetaxel for previously treated NSCLC cancer patients with KRAS G12C mutation, the progression-free survival for sotorasib compared with docetaxel was 5.6 months versus 4.5 months; hazard ratio. 0.66; *p* = 0.001) [5].

Fulzerasib (IBI351 or GFH925) is another KRAS-GDP inhibitor that showed an objective response rate of 49.1% and a median progression-free survival of 9.7 months in KRAS G12C-mutant NSCLC patients who had also failed previous treatments. The median overall survival is not yet available at the time of submission of this publication due to the still short median follow-up time [6]. The moderate, short-lived benefit of sotorasib, fulzerasib or adagrasib monotherapy [7] is due to the fact that epidermal growth factor receptor (EGFR) signaling is critical in KRAS G12C-mutant tumors. Consequently, the combination of a KRAS G12C inhibitor together with an EGFR tyrosine kinase inhibitor (erlotinib or gefitinib) has synergistic effects, as mutated KRAS is activated by upstream EGFR [8, 9]. When H358 cells (heterozygous for KRAS G12C) are stimulated with EGF (50 ng/mL), the level of GDP-bound KRAS G12C decreases. The combined inhibition of EGFR and KRAS leads to high levels of apoptosis, mitigating upstream KRAS activation [8]. Moreover, in KRAS G12C colorectal cancer models, EGFR signaling is the dominant mechanism of resistance to KRAS G12C inhibitors. Cetuximab (EGFR monoclonal antibody) sensitizes KRAS G12C colorectal cancer cell lines to sotorasib, and co-treatment with EGFR and KRAS G12C inhibitors reverts resistance to anti-EGFR antibodies [10]. While adding cetuximab to chemotherapy provided only modest improvements in overall survival compared to chemotherapy alone (median 11.3 months versus 10.1 months) [11], fulzerasib in combination with cetuximab in KRAS G12C metastatic NSCLC patients achieved an objective response rate of 66% and a median progression-free survival of 12.8 months. Overall survival data are still not available (KROCUS ClinicalTrials.gov number, NCT05756153) [12].

To further understand mechanistically whether EGFR signaling and extracellular signal-regulated kinase (ERK) rebound were also bona fide in KRAS G12C lung cancer cell lines, we investigated potential biomarkers and the synergism of sotorasib or fulzerasib with cetuximab. Some evidence suggested that receptor tyrosine kinase-RAS signaling activates MRAS, which forms a trimeric complex MRAS:SHOC2:PP1C that functions to reactivate ERK [13, 14]. In KRAS G12C NSCLC cells (H358 and LU65) treated with 1 µM sotorasib, MRAS and YAP1 were upregulated at 72 h, as observed in Western blot results [15]. Mitogen induced gene 6 (MIG6, also known as RALT or Gene 33) encoded by ERRB receptor feedback inhibitor 1 (ERRFI1) is a negative regulator of EGFR [16]. In oncogene-driven cancers, decreased MIG6 activates EGFR as a bypass signaling-mediated mechanism of resistance [17]. It was demonstrated that MIG6 protein level was reduced by sotorasib, and MIG6 knockdown reduced cell viability in the H358 cell line [17]. Similar to KRAS G12C colorectal cancer cell lines that express higher basal ERBB family expression [10], epithelial-like KRAS G12C NSCLC cells have a higher tendency to depend on ERBB signaling for survival under KRAS inhibition [18]. It was also identified that MRTX1133 (KRAS G12D inhibitor) in colorectal cancer cells promoted EGFR reactivation through downregulating MIG6 (ERRFI1) [17]. Concurrent treatment with EGFR inhibitors (cetuximab or gefitinib) and MRTX1133 produced a strong synergism in LS174T(KRAS G12D-mutant) cancer cells [17]. It was initially reported that MIG6 (encoded by ERRFI1) blocks EGFR activity by clamping onto its kinase domain, decreasing EGFR signaling [16], and also directing EGFR to lysosomes for degradation, resulting in a long-lasting inhibitory effect [19]. Of further interest is the fact that in a murine study of KRAS-mutant lung cancers, the presence of co-mutations in TP53 or STK11 (LKB1) impaired response to docetaxel and selumetinib (MEK inhibitor) when STK11 co-occurs as a mutation [20]. Henceforth, in the current study, we were interested in exploring the effect of fulzerasib and sotorasib not only as single agents but also in combination with cetuximab in KRAS G12C NSCLC cell lines, H358, H23 (STK11 and KEAP1 co-mutations) and H2030 (STK11 co-mutations).

Moreover, argininosuccinate synthase 1 (ASS1) encodes the enzyme that catalyzes arginosuccinate formation from citrulline to aspartate, the rate-limiting step in urea synthesis, and aberrant activation of Akt has been identified due to ASS1 loss when p53 is disabled [21]. Recently, in KRAS-mutant NSCLC, urea cycle reprogramming was observed as a common phenomenon associated with low ASS1 expression. Given that ASS1 downregulation is linked to poor prognosis in multiple tumors [22], we examined ASS1 behavior in KRAS G12C NSCLC cell lines treated with fulzerasib or sotorasib, alone or in combination with cetuximab. Paradoxically, the KRAS G13D mutation oppositely upregulated ASS1 in colorectal cancer, augmenting production of the metabolite fumarate [23]. This finding prompted us to further investigate fumarate dynamics before and after therapy, aiming to unveil a potential biomarker for surveillance in KRAS G12C-mutant NSCLC. Our findings reveal that in H358 cells, the combination of sotorasib or fulzerasib with cetuximab was synergistic, but the antitumor growth effect was not perceptible in H23 and H2030 cell lines.

## Results

### Fulzerasib and sotorasib demonstrate broad efficacy across KRAS G12C cell lines, even under EGFR-activating conditions

First, we tested a panel of KRAS G12C-mutant cell lines (*n* = 9) selected for our study against two KRAS G12C inhibitors, sotorasib and fulzerasib. Among them, seven were derived from lung adenocarcinoma, and one from pancreatic adenocarcinoma (MIA PaCa-2).

Culture conditions included full serum (10% FBS), serum starvation (0.5% HS), and serum starvation supplemented with exogenous hEGF (10 ng/mL). Under 10% FBS conditions (Supplementary Fig. 1), sotorasib exhibited a wide range of potencies (Table 1). H358 cells were highly sensitive (IC50 < 5 nM), while H23 and MIA PaCa-2 had higher IC50 values (10214 nM and 3514 nM, respectively). H2030 and non-KRAS G12C cells were largely resistant (IC50 > 25000 nM). Fulzerasib showed similar effectiveness, with moderately sensitive cell lines such as H23 (2470 nM) and MIA PaCa-2 (2907 nM), while remaining ineffective in H2030. H358, again, showed high sensitivity to fulzerasib (6.3 nM).

**Table 1:** .IC50 values of sotorasib and fulzerasib in KRASG12C and non-KRASG12C cell lines.

Inhibitor	Cell line	IC_50_ (nM) 10%FBS without hEGF	IC_50_ (nM) 0.5%HS without hEGF (10 ng/mL)	IC50 (nM) 0.5%HS with hEGF (10 ng/mL)
Sotorasib	H23	10,214.0	12,500^a^	>25,000^a^
	H358	<5.0	<5.0^a^	85.1^a^
	H2122^b^	7.1	612.5^a^	>25,000^a^
	H1792	55.6	9731.5	11,024.8
	MIA PaCa-2	3513.8	9002.0	15,644.0
	H2030	>25,000	13,763.9	23,296.8
	A549	>25,000	-	-
	H1819	>25,000	-	-
	SK-MES-1	>25,000	-	-
Fulzerasib	H23	2470.0	1597.0	2178.0
	H358	6.3	<5.0	28.4
	H2122^b^	33.6	<5.0	52.0
	H1792	50.9	1270.0	6067.0
	MIA PaCa-2	2907.0	8163.0	9578.0
	H2030	>25,000	13,143.0	>25,000
	A549	19,356.0	-	-
	H1819	19,427.4	-	-
	SK-MES-1	>25,000	-	-

^a^Values obtained from the paper [1].

^b^H2122 were cultured in 2% HS [24].

When cultured in 0.5% HS without hEGF (Supplementary Fig. 2), fulzerasib exhibited nanomolar to low micromolar efficacy in nearly all cell lines except H2030 (Table 1). The addition of hEGF to serum-starved media showed notable resistance to sotorasib in multiple lines. H23, H2122, and H2030 all exhibited IC50 values exceeding 23,000 nM. In contrast, fulzerasib maintained significantly lower IC50 values in the same lines: 2178 nM in H23 and 52.0 nM in H2122.

### Combination of fulzerasib or sotorasib with cetuximab exhibits general synergistic effects across most KRAS G12C-mutant cell lines

Next, we assessed the effectiveness of the dual combination of sotorasib/fulzerasib with cetuximab. CI values were calculated using the Chou–Talalay method, where a CI < 1 indicates synergy, CI = 1 indicates additivity, and CI > 1 indicates antagonism. Preliminary experiments revealed no observable effects of drug combinations under full serum conditions (10% FBS). Subsequently, serum-starvation media supplemented with hEGF were used to activate EGFR signaling. This experimental approach has been adopted in other similar studies [24].

**Fig. 1:**
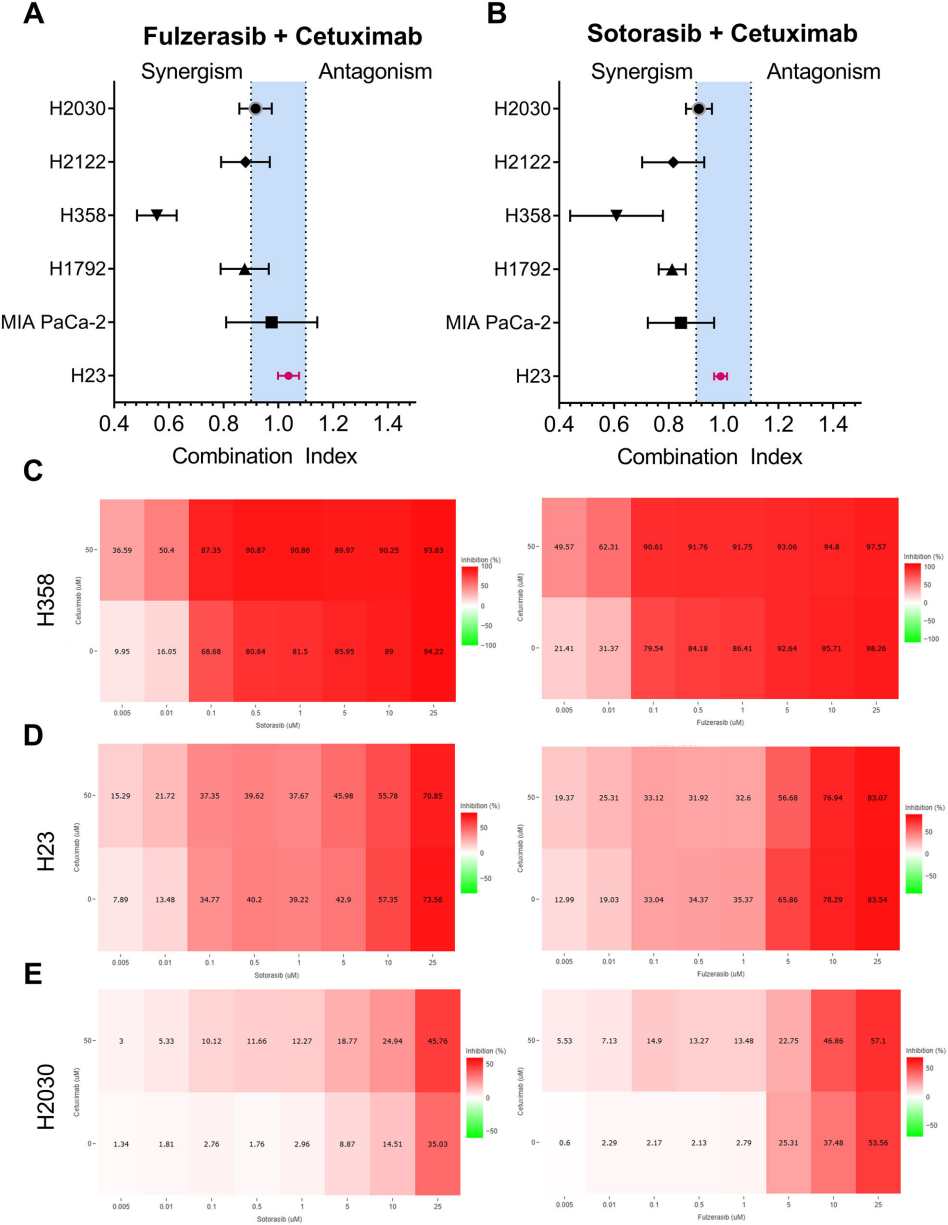
Effects of sotorasib/fulzerasib combined with cetuximab in KRAS G12C mutant cell lines. Data was obtained at 72 h by MTT. Culture medium consisted of RPMI + 0.5% HS with 10 ng/ml of hEGF, except for H2122, which was cultured with 2% HS supplemented with hEGF. **A** Sotorasib + Cetuximab **B** Fulzerasib + Cetuximab. Values shown are means ± SD, represented with a horizontal bar, from three independent experiments. Data was analyzed by Chou–Talalay method, indicating CI < 1 synergy, CI = 1 additivity, and CI > 1 antagonism. Drug combination inhibition matrix of sotorasib (left), fulzerasib (right) and their combination with cetuximab on **C** H358, **D** H23 and **E** H2030 cultured in RPMI + 0.5% HS with 10 ng/ml of hEGF. The first row represents inhibition of cell proliferation in combination of fulzerasib/sotorasib at different doses with cetuximab (50 µg/mL), the second row shows inhibition of cell proliferation on a single agent of fulzerasib/sotorasib. The intensity of the red color increases with greater inhibition of cell proliferation. Values shown are means of three independent experiments.

In the panel treated with fulzerasib plus cetuximab, only H358 showed a robust synergistic effect, i.e., CI value < 1 (Fig. 1A). Notably, H2030, H2122, MIA PaCa-2, and H1792 cell lines exhibited values within the threshold between synergism and additive effect. H23 cells, in contrast, show a complete additive effect. No antagonism was observed with the fulzerasib and cetuximab combination in any cell line tested. Similarly, the combination of sotorasib plus cetuximab showed comparable results. Strong synergy was again observed in H358 cells (Fig. 1B). H2030, H2122, MIA PaCa-2, and H1792 cells exhibited slight synergistic effects, while H23 remained additive. Notably, the combination of cetuximab with sotorasib antagonism was not observed for the three cell lines.

The inhibitory effects of sotorasib and fulzerasib could be represented in drug combination inhibition matrices (Fig. 1C–E). For H358 cells, both sotorasib and fulzerasib per se induced substantial inhibition of cell proliferation, which was enhanced by the addition of cetuximab, indicating a synergistic interaction. For H23 cells, treatment with either inhibitor alone resulted in partial inhibition, with no improvement upon co-treatment with cetuximab. In contrast, H2030 cells exhibited resistance to sotorasib and fulzerasib as single agents. However, the combination of cetuximab increased the inhibitory effect.

### Combination with cetuximab mitigates MAPK pathway reactivation induced by fulzerasib/sotorasib monotherapy

The experiments presented so far established the effectiveness of fulzerasib and sotorasib in vitro, which increased when combined with cetuximab in our panel of KRAS G12C-mutant cell lines. Next, we used Western blotting to analyze the effects of these inhibitors on key signal transduction proteins of the RAF/MEK/ERK and phosphoinositide 3-kinase (PI3K) pathways (initiators of resistance mechanisms and potential biomarkers of cancer progression) in three cell line models according to their different sensitivities to fulzerasib and sotorasib. H358, H23 and H2030 cells were treated with fulzerasib, sotorasib or cetuximab alone, and with fulzerasib or sotorasib in combination with cetuximab. Samples were obtained at basal levels, 24 h, 48 h and 72 h. The intensity of protein bands was quantified relative to untreated controls (set as 1.00) and normalized with the loading control Hsp90.

**Fig. 2:**
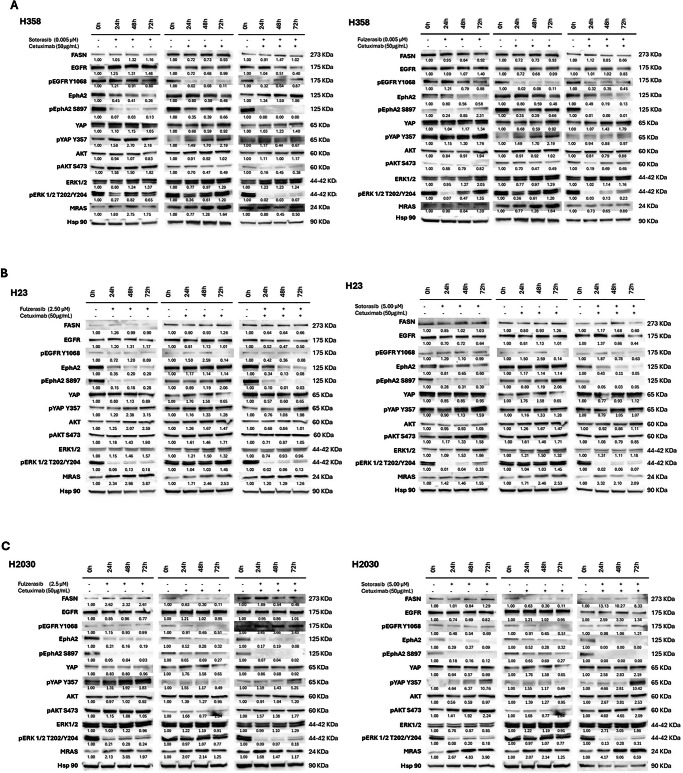
Western blot analysis of sotorasib, fulzerasib, cetuximab and sotorasib/fulzerasib combined with cetuximab. Culture medium consisted of RPMI + 10% FBS, and incubation time was 0 h, 24 h, 48 h and 72 h. Drug concentration was established according to cell lines IC50 and maximum plasma concentration observed in patients. Fulzerasib, 0.005 µM for H358 and 2.50 µM for H23 and H2030. Sotorasib, 0.005 µM for H358 and 5 µM for H23 and H2030. Cetuximab was tested in all cases at 50 µg/mL. **A** H358 **B** H23 and **C** H2030 cell lines. The intensity of the bands was first normalized using Hsp90 and then normalized to the basal condition (timepoint 0). Bands were quantified using ImageJ software.

Levels of EGFR did not change with treatment or over time in most of the cell lines. pEGFR Y1068 was observed to increase at 72 h following treatment with KRAS G12C inhibitors and their combination, but not with cetuximab alone, in H358 and H2030 cells (Fig. 2A, B, C). Interestingly, the activated membrane receptor erythropoietin-producing hepatocellular (Eph)A2 (EphA2) S897 levels were reduced by KRAS G12C inhibitors in all cell lines. The indicator of resistance initiation, pYAP Y357, increased to a greater extent with sotorasib/fulzerasib or cetuximab alone in H358 cells, but was reduced by the combination. Similarly, in H23 cells, pYAP Y357 showed a slight increase across all treatments. On the other hand, H2030 cells exhibited a strong increase in pYAP Y357 upon KRAS G12C inhibitor monotherapy and combination treatments, but not with cetuximab alone. KRAS activation is known to drive oncogenic malignancy, mainly via two downstream pathways regulated by ERK and AKT. Total AKT levels remained stable across all three cell lines. However, in H358 cells, pAKT S473 levels decreased in all cases except with sotorasib treatment. Regarding H23 cells, pAKT S473 increased with most treatments, except when KRAS G12C inhibitors were combined with cetuximab. Reactivation of the MAPK pathway was observed through pERK ½ T202/Y204 at 48–72 h in all cell lines. H358, the most sensitive cell line with a lower IC50, showed a clear relapse of pERK 1/2 T202/Y204 upon sotorasib/fulzerasib treatment. This relapse was delayed when KRAS G12C inhibitors were combined with cetuximab. The same behavior was observed in H23 and H2030 cells. In all cell lines, the combination delayed the appearance of pERK 1/2 T202/Y204. MRAS levels increased in H358 cells upon cetuximab treatment, but not with either KRAS G12C inhibitor alone. In H23 cells, MRAS levels increased upon fulzerasib treatment; however, this increase was more pronounced in H2030 cells treated with sotorasib or with the sotorasib-cetuximab combination.

### Distinct protein expression patterns reveal MIG6 as a potential biomarker

**Fig. 3:**
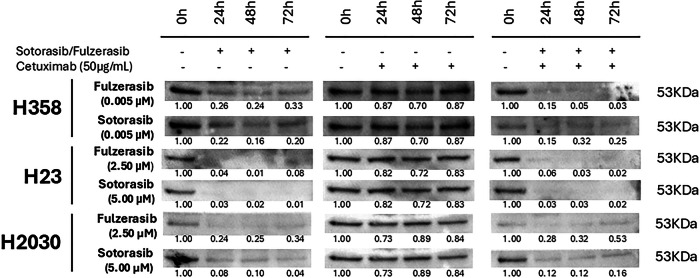
Sotorasib and fulzerasib reduce MIG6 expression in mutant-KRAS G12C cell lines. Cells were treated with fulzerasib, sotorasib, cetuximab and sotorasib/fulzerasib combined with cetuximab. Fulz, fulzerasib, 0.005 µM for H358 and 2.5 µM for H23 and H2030. Soto, sotorasib, 0.005 µM for H358 and 5 µM for H23 and H2030. Cetuximab was tested in all cases at 50 µg/mL. Loading control Hsp90 corresponds to bands of Fig. 2. The intensity of the bands was first normalized using Hsp90, and then normalized to the basal condition (timepoint 0). Bands were quantified using ImageJ software.

In parallel, we carried out a study on new biomarkers. Literature research revealed MIG6 as a key regulator of EGFR activation [25]. Therefore, we investigated the effects of sotorasib/fulzerasib and their respective combinations with cetuximab on MIG6 levels. A similar pattern was observed in H358, H23 and H2030 cells at basal levels, in which MIG6 is present, but its levels reduced dramatically upon sotorasib/fulzerasib treatment (Fig. 3). This reduction was observed at 24 h, and levels did not increase during the next 72 h. Similar results were obtained in combination of cetuximab with the KRAS G12C inhibitors. However, cetuximab alone did not change MIG6 levels in any cell line.

### Differential fumarate and ASS1 profiles reveal metabolic predictors of KRAS G12C inhibitor sensitivity

**Fig. 4:**
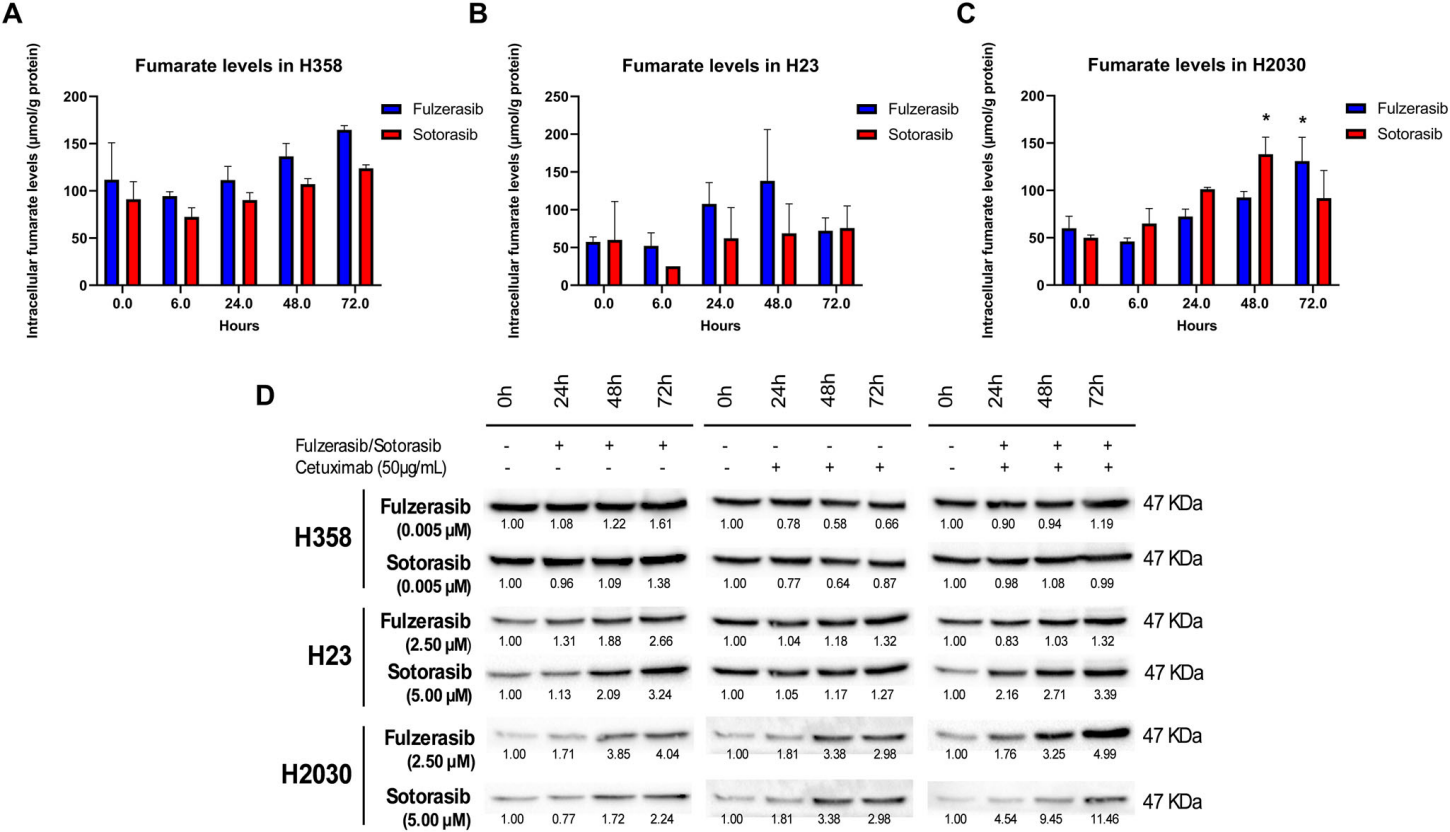
Effects of KRAS G12C inhibitors on fumarate accumulation and ASS1 levels in KRAS G12C-mutant cell lines. Fumarate accumulation curves at 72 h upon fulzerasib/sotorasib treatment in **A** H358, **B** H23 and **C** H2030 cell line. Bars indicate mean ± SEM of two independent experiments. *p < 0.05. Values shown are means of two independent experiments. **D** Western blot analysis of ASS1 upon treatment in KRAS G12C-mutant cell lines (H358, H23 and H2030). Loading control Hsp90 corresponds to bands of Fig. 2. The intensity of the bands was first normalized using Hsp90, and then normalized to the basal condition (timepoint 0). Bands were quantified using ImageJ software.

Fumarate, a metabolic intermediate in the tricarboxylic acid cycle, has been reported to influence tumor progression [23]. In this context, we quantified fumarate levels in three cell lines (Fig. 4A–C). In H358 cells treated with sotorasib or fulzerasib, basal levels were approximately 150 µmol of fumarate/g of protein. These levels decreased at 6 h but gradually increased, reaching a maximum at 72 h (Fig. 4A). H23 cells showed lower basal fumarate levels and distinct patterns in response to sotorasib and fulzerasib treatment. However, both KRAS G12C inhibitors reached their maximum at 72 h (Fig. 4B). H2030 exhibited a different pattern with sotorasib treatment, increasing its levels from the basal state up to a maximum at 48 h, and afterwards, decreasing slightly at 72 h. For fulzerasib, the levels were increased basally, decreasing at 6 h and increasing to a maximum at 72 h (Fig. 4C).

We also found evidence in literature that ASS1, a metabolic enzyme involved in the urea cycle and linked to fumarate metabolism, has been associated with a favorable prognosis when expressed at high levels [26]. The most sensitive cell line to sotorasib/fulzerasib treatment did not show a considerable increase, either in monotherapy or in combination with cetuximab (Fig. 4D). In contrast, the most resistant cell lines, H23 and H2030, showed low basal levels that increased in a time-dependent manner upon KRAS G12C inhibitor monotherapy and in combination with cetuximab.

### Fulzerasib and cetuximab synergistically suppress KRAS G12C lung tumor growth in mice

To determine the antitumor efficacy of the combination fulzerasib with cetuximab in vivo, we implanted 2 × 10⁶ human lung cancer KRAS G12C-mutant H358 cells subcutaneously into the right flank of BALB/c nude mice. Mice were randomized into 10 groups (*n* = 5 per group), and administered with vehicle orally and intravenously, fulzerasib at 0.5 mg/kg or 1 mg/kg orally and cetuximab at 0.3 mg/kg or 1 mg/kg intravenously at single agent and in combinations of both drugs for 21 days.

**Fig. 5:**
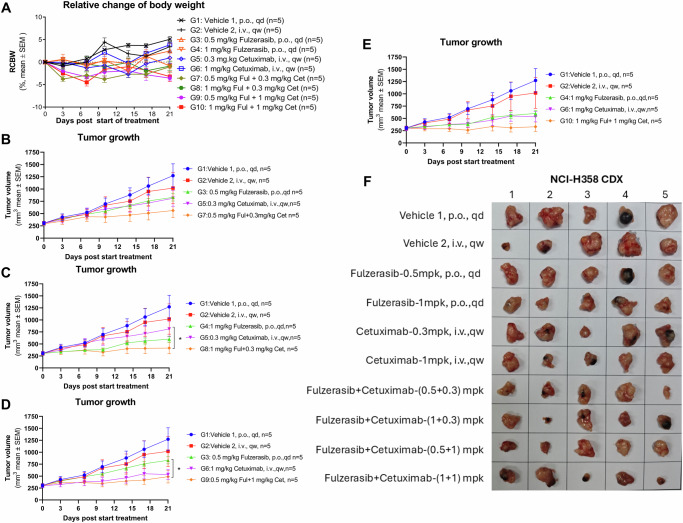
Combination treatment with fulzerasib and cetuximab reduces tumor growth in H358 xenografts. **A** Relative change in body weight (RCBW) over the treatment period in mice (*n* = 5 per group) shows tolerability across all dosing regimens (mean ± SEM). **B**-**E** Tumor volumes in mice, measured by caliper, are shown as mean ± SEM. Graphs are grouped by concentration; combinations are shown comparatively. The number of mice per group is indicated in the plots. Data was analyzed by two-way ANOVA followed by a Tukey’s multiple comparison test, *p < 0.05. **F** Representative tumor images collected at the end of the study.

No considerable difference in body weight was observed between groups over the treatment period, with no decrease surpassing 5.0% in any case (Fig. 5A). No mortality was observed in the in vivo experiments. At the study endpoint, fulzerasib and cetuximab as single agents demonstrated considerable tumor growth inhibition, showing greater efficacy at high concentrations. It should be emphasized that the combination of fulzerasib with cetuximab synergistically enhanced tumor growth inhibition, reaching 97% inhibition at the highest concentrations of both agents (Fig. 5B). Unfortunately, serial tumor sections were not available for additional staining; therefore, Ki67, vimentin, and caspase-3 cleavage were not examined in these tumors.

## Discussion

KRAS G12C-mutant advanced or metastatic NSCLC portends an ominous prognosis. Improvement has been recently achieved with allele-specific KRAS G12C inhibitors that lock the KRAS G12C protein in the GDP-bound inactive state. However, the first-class inhibitors sotorasib and adagrasib attained responses in only 41%-46% of cases and with a short median progression-free survival of 6.3 months and 6.5 months, respectively [27]. This led to the approval of sotorasib and adagrasib as a second-line treatment for KRAS G12C NSCLC patients [28, 29].

Since we initiated the current study, there has been a surge of novel RAS inhibitors with activity against the GTP-bound “ON” versus GDP-bound “OFF” forms of RAS, as well as pan-KRAS inhibitors. Many are being developed in clinical trials, as noted in a recent review [30]. Fulzerasib (GFH925) shows high in vitro potency and selectivity, favorable pharmacokinetic profiles across species, and significant in vivo antitumor efficacy in diverse cancer-related xenograft models, including intracranial tumors [31].

A main mechanism of resistance to KRAS G12C inhibitor monotherapy is the reactivation by upstream feedback EGFR signaling that shifts KRAS G12C to its GTP-bound state, associated with the recovery of ERK phosphorylation downstream of KRAS [8, 32]. The increased EGFR signaling shifted KRAS G12C toward the GTP-bound active state, rendering GDP-state inhibitors, such as the preclinical inhibitor ARS-1620, unable to efficiently block ERK signaling through KRAS G12C [33]. Moreover, following receptor tyrosine kinase activation, MRAS, a neighboring member of the RAS family, is activated [13, 14, 34]. Recently, in H358 and LU65 KRAS G12C-mutant lung cancer cells treated with 1 µM sotorasib, overexpression of MRAS and CYR61 (a readout of YAP1) was observed 72 h later, as determined by Western blot analysis [15]. Henceforth, in our study, we were interested in examining the occurrence of reactivation of ERK as well as MRAS and YAP1 signaling, principally in H358, H23, and H2030 KRAS G12C cell lines that exhibit distinct sensitivity to fulzerasib and sotorasib.

The findings obtained herein show that cetuximab, as an EGFR inhibitor, enhances the potency of KRAS G12C inhibitors, such as fulzerasib, in vitro in H358 cells, and extends the survival of mice bearing subcutaneous H358 xenografts. Cotreatment with fulzerasib and cetuximab suppressed the expression of ERK and AKT, as well as that of MRAS and YAP1 Y357. Phosphorylation of YAP1 at tyrosine 357 indicates its nuclear localization and activation. Moreover, EphA2 expression was down-regulated as well. EphA2 expression has been reported to be increased in KRAS-mutant NSCLC patients and to predict poorer progression-free and overall survival [35]. Of interest is the fact that EphA2 is a transcriptional target of RAS-ERK signaling [36]. EphA2 phosphorylation at serine 897 by AKT has also been reported [37]. KRAS activates lipogenesis, and specific induction of fatty acid synthase (FASN) has been observed in KRAS-mutant NSCLC cell lines [38]. Intriguingly, EphA2 overexpression in HER2-positive breast cancers activates the tricarboxylic acid (TCA) cycle and FASN [39]. FASN could serve as a biomarker predicting response to KRAS G12C inhibitors as well as to immune checkpoint inhibitors, since lower expression of FASN has recently been shown to correlate with a higher percentage of cytotoxic CD8 + T cells in hepatocellular carcinoma [40]. In addition, also in hepatocellular carcinoma, FASN reduces constitutive overexpression of PD-L1 by abolishing its post-translational palmitoylation [41]. Sotorasib replicated the effects of fulzerasib in H358 cells (Fig. 2A).

With the exception of H358 cells, the combination of fulzerasib with cetuximab did not result in the death of KRAS-mutant NSCLC cells in vitro, particularly in the H2030 cell line, where YAP1 Y357 reactivation was observed at 72 h and was not ablated by the combination with cetuximab. Treatment with sotorasib and cetuximab induced higher overexpression of YAP1 Y357 and MRAS in Western blot analysis compared with fulzerasib and cetuximab (Fig. 2C). The combination of fulzerasib and cetuximab produced only additive synergism in vitro in the H23 cell line, where the upregulation of MRAS and YAP1 was present but more moderate than in the H2030 cell line (Fig. 2C).

These results led us to interpret that H358 cells epitomize a subgroup of KRAS G12C NSCLC patients for whom the combination of fulzerasib plus cetuximab has demonstrated a response rate of 68.9% with a median progression-free survival of 12.5 months and a good safety profile [12]. It is tempting to speculate that clinical outcomes may be better for patients whose tumors behave similarly to H358 cells. Therefore, combining selective GDP-bound state active KRAS G12C inhibitors with EGFR inhibitors such as cetuximab could provide a superior therapeutic benefit.

The development of biomarkers, especially YAP1 and MRAS, to predict response to co-treatment of KRAS G12C-mutant NSCLC with EGFR inhibitors is warranted. Intuitively, a substantial proportion of patients could behave similarly to the H2030 cell line, being rather de novo KRAS-independent, in whom YAP1 upregulation occurred regardless of combination therapy with EGFR blockade. In addition, it seems that in the relatively resistant or refractory KRAS G12C cell models H23 and H2030, FASN and EphA2 are abolished. Co-treatment with YAP inhibitors has been reported to overcome innate or acquired resistance to KRAS G12C inhibitors [42]. We were compelled to investigate MIG6, since it was previously identified as a negative inhibitor of EGFR signaling [16] and as a mediator of adaptive and acquired resistance to ALK and ROS1 kinase inhibitors [17]. Importantly, MRTX1133 (KRAS G12D inhibitor) downregulates MIG6 expression in KRAS G12D-mutant colorectal cancer cell lines and activates EGFR, leading to RAS-ERK signaling reactivation, suggesting that EGFR inhibitors may be beneficial in KRAS G12C-mutant NSCLC. The anti-growth effects of various drug pairs showed that MEK, mTOR, PI3K, SHP2, and CDK4/6 inhibitors had modest effects in combination with MRTX1133. In contrast, concurrent treatment with EGFR inhibitors (cetuximab or gefitinib) and MRTX1133 produced strong synergism [43]. We noted that fulzerasib and sotorasib similarly reduced MIG6 expression in all three KRAS G12C cell lines (Fig. 3). Although the reduction was comparable across all these models, as described above, MIG6 downregulation by KRAS G12C inhibition predicts the need for simultaneous EGFR inhibition only in the H358 cell line.

Moreover, we were prompted to examine the expression of arginosuccinate synthase (ASS1), which was recently reported to be downregulated in KRAS-mutant NSCLC [26]. Downregulation of ASS1 is associated with poor prognosis [22]. Notably, fulzerasib and sotorasib upregulated ASS1 expression in all three cell lines, with further increases observed when combined with cetuximab in H23 cells and even more so in H2030 cells (Fig. 4D). Accordingly, fumarate levels were measured, and their upregulation was noted in the three cell lines, most prominently at 72 h, following treatment with either fulzerasib or sotorasib.

Our data help demonstrate that EGFR inhibitors may represent a promising combination strategy with KRAS G12C. However, the present study cannot demonstrate that either MIG6 or ASS1, although potentially important, can reliably distinguish KRAS G12C-sensitive models that respond to co-treatment with KRAS G12C and EGFR inhibitors. Further research is warranted to clarify the role of ASS1 expression and fumarate levels. Notwithstanding, these markers should be considered in translational research trials. Efforts to develop methods to gauge and monitor the expression of MRAS and YAP1 are highly warranted.

## Materials and methods

### Cell lines and drugs

The cell lines were purchased from ATCC. H23, H2030, H358, H2122, MIA PaCa-2, H1792, A549, H1819 and SK-MES-1 were cultured in RPMI medium (Gibco, Thermo Fisher Scientific, Waltham, MA), supplemented with 10% fetal bovine serum (Gibco) and 1% penicillin/streptomycin (Gibco) and L-glutamine (Gibco), at 37 °C in humidified incubators with 5% CO_2_ atmosphere. Mycoplasma testing of these cell lines is conducted regularly with a commercially obtained mycoplasma detection kit (Minerva Biolabs). Fulzerasib was kindly provided by Genfleet (Shanghai); Sotorasib and cetuximab were purchased from Selleck Chemicals (Houston, TX).

### MTT assay

The Thiazolyl Blue Tetrazolium Bromide (MTT) assay was used to assess the effects of the drugs in terms of cell viability/proliferation. In this assay, 2000 cells (for H23, H358, H2030, H1792, MIA PaCa, A549), 4000 cells (for H1819 and SK-MES-1) and 8000 cells (for H2122) were seeded in 96-well plates. The cells were allowed to attach for 24 h.

To evaluate the impact of KRAS inhibitors alone, the cells were then treated with sotorasib or fulzerasib in RPMI supplemented with 10% FBS. Regarding the combinatory assays, the cells were treated with fulzerasib or sotorasib with cetuximab in RPMI supplemented with 0.5% human serum (HS) (Gibco) or supplemented with 10 ng/mL of hEGF (Gibco) except for the H2122 cells, which were treated in RPMI + 2% HS and hEGF supplemented. Human EGF addition was restricted to the combination of drugs with cetuximab in cell viability assays.

At the end of the incubation period, adherent cells were incubated with medium containing 0.75 ng/mL Thiazolyl Blue Tetrazolium Bromide (MTT, Sigma Aldrich, St Louis, MO). Formazan crystals in viable cells were solubilized in 100 µL DMSO (Merck, Darmstadt, Germany), and cell viability was quantified by reading the A_565_ using an Infinite M Plex Tecan microplate reader (Männedorf, Switzerland). Data were derived from at least three independent experiments, normalized with the A_565_ obtained for control cells (growing in the absence of drugs or hEGF) and presented as mean ± SEM.

### Western blotting

Subconfluent cultures were treated in T-75 flasks with inhibitors in RPMI + 10% FBS. Cells were washed twice with cold PBS 1X (Gibco) and lysed in RIPA buffer (20 mM Tris–HCl pH 7.5, 150 mM NaCl, 1 mM Na_2_EDTA, 1 mM EGTA, 1% NP-40, 1% sodium deoxycholate, 2.5 mM sodium pyrophosphate, 1 mM β-glycerophosphate, 1 mM Na_3_VO_4_, 1 µg/ mL leupeptin (Cell Signaling Technologies, Beverly, MA), 2 mM PMSF and Protease Inhibitor Cocktail (Roche Diagnostics, Mannheim, FRG) and passed through an insulin syringe. Lysates were centrifuged at 14000 rpm at 4 °C and quantified using the BCA Method. Protein sample concentrations were adjusted to the same level.

Protein extracts (20–40 µg) were boiled in Laemmli buffer (NuPAGE- LDS sample buffer 4X; Invitrogen), resolved in SDS-polyacrylamide gels and transferred to PVDF membranes (Bio-Rad, Hercules, CA). Membranes were incubated for 1 h in 5% Non-fat dry milk in TBS 1X (Bio-Rad), cut, incubated with primary antibodies (Supplementary Table 1) o/n at 4 ^◦^C, washed three times with PBS-Tween 0.1% and incubated for 2 h with a secondary antibody (Supplementary Table 1). Finally, membranes were washed with PBS-Tween 0.1%, revealed with Supersignal Chemiluminiscence substrate (Thermofisher, Waltham, MA) and read with a Bio-Rad ChemiDocMP Imaging System. Bands obtained were quantified using ImageJ software. The intensity of the bands was normalized using Hsp90.

### Fumarate levels

Subconfluent cultures were treated in T-25 flasks with inhibitors in RPMI + 10% FBS. A spectrophotometric kit (MAK492, Sigma-Aldrich) was used to test fumarate levels. Cells were washed twice with cold PBS 1X and lysed in fumarate buffer provided in the kit. Lysates were cleared by centrifugation at 14,000 rpm at 4 °C and quantified using the BCA method. The procedure was followed according to the manufacturer’s instructions. Fumarate levels were represented in µmol of fumarate/g protein.

### In vivo experiments

Female, BALB/c nude mice, 6–8 weeks at the time of inoculation, were inoculated with 2 × 10^6^ NCI-H358 cells in 50% Matrigel (0.2 mL/mouse) subcutaneously in the right flank of the animals. Treatments were started 21 days after tumor inoculation, and mice were randomly assigned to 10 groups, each comprising 5 mice. Fulzerasib was administered orally, and cetuximab intravenously. Vehicle group for fulzerasib received 5%DMSO + 10% Solutol HS 15 + 85% (6%HP-β-CD in water) orally once per day. The vehicle group for cetuximab received PBS intravenously once per week. The technician was blinded to the treatment assignments.

Sample size was considered adequate to detect biologically relevant differences between groups while minimizing animal use in accordance with ethical guidelines. Two cases were defined to exclude animals from the study. Animal deaths are defined by a relative change of body weight exceeding—20% or >2000 mm^3^ tumor volume. Tumor growth exceeded twofold compared with pre-dosing levels. No animals met the predefined exclusion criteria. Experiments were performed at PharmaLegacy, Shanghai, China.

### Statistical analysis

GraphPad Prism 9.0 was used for statistical analysis. All values are given as mean ± SD or SEM as indicated in figure legends; *p*-values of <0.05 were considered statistically significant. Comparisons between two groups were made by Student’s *t* test. Synergism between two drugs was made by the Chou–Talalay method. In the in vivo *testing*, for comparisons of more than two groups, we used ANOVA followed by Tukey’s multiple comparison test. Significance was defined when *p*-value < 0.05. No statistical method was used to predetermine sample size in animal studies. Comparisons of basal fumarate levels and levels at progression upon treatment were analyzed using a one-way ANOVA followed by Dunnett’s multiple comparison test. All data is adjusted to meet the test’s assumptions.

## Supplementary information

Revised Supplementary information-Clean version

Original data

## Data Availability

The datasets analyzed during the current study are available from the corresponding author on reasonable request. All relevant data supporting the findings of this study are included within the article and its Supplementary Information files.

## References

[1] Adachi Y, Ito K, Hayashi Y, Kimura R, Tan TZ, Yamaguchi R, et al. Epithelial-to-mesenchymal transition is a cause of both intrinsic and acquired resistance to the KRAS G12C inhibitor in KRAS G12C-mutant non-small cell lung cancer. Clin Cancer Res. 2020;26:5962–73. 32900796 10.1158/1078-0432.CCR-20-2077

[2] Mitsudomi T, Steinberg SM, Oie HK, Mulshine JL, Phelps R, Viallet J, et al. Ras gene mutations in non-small cell lung cancers are associated with shortened survival irrespective of treatment intent. Cancer Res. 1991;51:4999–5002. 1654209

[3] Gray JE, Hsu H, Younan D, Suri G, Chia V, Spira A, et al. Real-world outcomes in patients with KRAS G12C-mutated advanced non-small cell lung cancer treated with docetaxel in second-line or beyond. Lung Cancer. 2023;181:107260. 37285629 10.1016/j.lungcan.2023.107260

[4] Skoulidis F, Li BT, Dy GK, Price TJ, Falchook GS, Wolf J, et al. Sotorasib for lung cancers with KRAS p.G12C mutation. N Engl J Med. 2021;384:2371–81. 34096690 10.1056/NEJMoa2103695 PMC9116274

[5] de Langen AJ, Johnson ML, Mazieres J, Dingemans AC, Mountzios G, Pless M, et al. Sotorasib versus docetaxel for previously treated non-small-cell lung cancer with KRAS(G12C) mutation: a randomised, open-label, phase 3 trial. Lancet. 2023;401:733–46. 36764316 10.1016/S0140-6736(23)00221-0

[6] Zhou Q, Meng X, Sun L, Huang D, Yang N, Yu Y, et al. Efficacy and safety of KRASG12C inhibitor IBI351 monotherapy in patients with advanced NSCLC: results from a phase 2 pivotal study. J Thorac Oncol. 2024;19:1630–9. 39127176 10.1016/j.jtho.2024.08.005

[7] Jänne PA, Riely GJ, Gadgeel SM, Heist RS, Ou SI, Pacheco JM, et al. Adagrasib in non-small-cell lung cancer harboring a KRAS(G12C) mutation. N Engl J Med. 2022;387:120–31. 35658005 10.1056/NEJMoa2204619

[8] Patricelli MP, Janes MR, Li LS, Hansen R, Peters U, Kessler LV, et al. Selective inhibition of oncogenic KRAS output with small molecules targeting the inactive state. Cancer Discov. 2016;6:316–29. 26739882 10.1158/2159-8290.CD-15-1105

[9] Lito P, Solomon M, Li LS, Hansen R, Rosen N. Allele-specific inhibitors inactivate mutant KRAS G12C by a trapping mechanism. Science. 2016;351:604–8. 26841430 10.1126/science.aad6204 PMC4955282

[10] Amodio V, Yaeger R, Arcella P, Cancelliere C, Lamba S, Lorenzato A, et al. EGFR blockade reverts resistance to KRAS(G12C) inhibition in colorectal cancer. Cancer Discov. 2020;10:1129–39. 32430388 10.1158/2159-8290.CD-20-0187 PMC7416460

[11] Pirker R, Pereira JR, Szczesna A, von Pawel J, Krzakowski M, Ramlau R, et al. Cetuximab plus chemotherapy in patients with advanced non-small-cell lung cancer (FLEX): an open-label randomised phase III trial. Lancet. 2009;373:1525–31. 19410716 10.1016/S0140-6736(09)60569-9

[12] Majem M, Gregorc V, Lo Russo G, Shan Y, Zhu H, Rosell R, et al. First-line (1L) fulzerasib (FUL) + cetuximab (CETU) in KRAS G12Cm advanced NSCLC: updated results from the KROCUS study. J Thoracic Oncol. 2025;20:20.

[13] Simanshu DK, Nissley DV, McCormick F. RAS proteins and their regulators in human disease. Cell. 2017;170:17–33. 28666118 10.1016/j.cell.2017.06.009 PMC5555610

[14] Kwon JJ, Hajian B, Bian Y, Young LC, Amor AJ, Fuller JR, et al. Structure-function analysis of the SHOC2-MRAS-PP1C holophosphatase complex. Nature. 2022;609:408–15. 35831509 10.1038/s41586-022-04928-2 PMC9694338

[15] Adachi Y, Kimura R, Hirade K, Yanase S, Nishioka Y, Kasuga N, et al. Scribble mis-localization induces adaptive resistance to KRAS G12C inhibitors through feedback activation of MAPK signaling mediated by YAP-induced MRAS. Nat Cancer. 2023;4:829–43. 37277529 10.1038/s43018-023-00575-2

[16] Zhang X, Pickin KA, Bose R, Jura N, Cole PA, Kuriyan J. Inhibition of the EGF receptor by binding of MIG6 to an activating kinase domain interface. Nature. 2007;450:741–4. 18046415 10.1038/nature05998 PMC3561764

[17] Chen N, Tyler LC, Le AT, Welsh EA, Fang B, Elliott A, et al. MIG6 mediates adaptive and acquired resistance to ALK/ROS1 fusion kinase inhibition through EGFR bypass signaling. Mol Cancer Ther. 2024;23:92–105. 37748191 10.1158/1535-7163.MCT-23-0218 PMC10762338

[18] Kitai H, Ebi H, Tomida S, Floros KV, Kotani H, Adachi Y, et al. Epithelial-to-mesenchymal transition defines feedback activation of receptor tyrosine kinase signaling induced by MEK inhibition in KRAS-mutant Lung cancer. Cancer Discov. 2016;6:754–69. 27154822 10.1158/2159-8290.CD-15-1377 PMC4957999

[19] Frosi Y, Anastasi S, Ballarò C, Varsano G, Castellani L, Maspero E, et al. A two-tiered mechanism of EGFR inhibition by RALT/MIG6 via kinase suppression and receptor degradation. J Cell Biol. 2010;189:557–71. 20421427 10.1083/jcb.201002032 PMC2867293

[20] Chen Z, Cheng K, Walton Z, Wang Y, Ebi H, Shimamura T, et al. A murine lung cancer co-clinical trial identifies genetic modifiers of therapeutic response. Nature. 2012;483:613–7. 22425996 10.1038/nature10937 PMC3385933

[21] Miyamoto T, Lo PHY, Saichi N, Ueda K, Hirata M, Tanikawa C, et al. Argininosuccinate synthase 1 is an intrinsic Akt repressor transactivated by p53. Sci Adv. 2017;3:e1603204. 28560349 10.1126/sciadv.1603204 PMC5438217

[22] Lim LQJ, Adler L, Hajaj E, Soria LR, Perry RB-T, Darzi N, et al. ASS1 metabolically contributes to the nuclear and cytosolic p53-mediated DNA damage response. Nat Metab. 2024;6:1294–309. 38858597 10.1038/s42255-024-01060-5 PMC11272581

[23] Doubleday PF, Fornelli L, Ntai I, Kelleher NL. Oncogenic KRAS creates an aspartate metabolism signature in colorectal cancer cells. FEBS J. 2021;288:6683–99. 34227245 10.1111/febs.16111 PMC8648997

[24] García-Roman S, Garzón-Ibáñez M, Bertrán-Alamillo J, Jordana-Ariza N, Giménez-Capitán A, García-Peláez B, et al. Vaccine antibodies against a synthetic epidermal growth factor variant enhance the antitumor effects of inhibitors targeting the MAPK/ERK and PI3K/Akt pathways. Transl Oncol. 2024;40:101878. 38183801 10.1016/j.tranon.2024.101878 PMC10818253

[25] Maity TK, Venugopalan A, Linnoila I, Cultraro CM, Giannakou A, Nemati R, et al. Loss of MIG6 accelerates initiation and progression of mutant epidermal growth factor receptor-driven lung adenocarcinoma. Cancer Discov. 2015;5:534–49. 25735773 10.1158/2159-8290.CD-14-0750 PMC4560174

[26] Gai X, Liu Y, Lan X, Chen L, Yuan T, Xu J, et al. Oncogenic KRAS induces arginine auxotrophy and confers a therapeutic vulnerability to SLC7A1 inhibition in non-small cell lung cancer. Cancer Res. 2024;84:1963–77. 38502865 10.1158/0008-5472.CAN-23-2095

[27] Singhal A, Li BT, O’Reilly EM. Targeting KRAS in cancer. Nat Med. 2024;30:969–83. 38637634 10.1038/s41591-024-02903-0 PMC11845254

[28] Hendriks LE, Kerr KM, Menis J, Mok TS, Nestlé U, Passaro A, et al. Oncogene-addicted metastatic non-small-cell lung cancer: ESMO clinical practice guideline for diagnosis, treatment and follow-up. Ann Oncol. 2023;34:339–57. 36872130 10.1016/j.annonc.2022.12.009

[29] Meyer ML, Fitzgerald BG, Paz-Ares L, Cappuzzo F, Jänne PA, Peters S, et al. New promises and challenges in the treatment of advanced non-small-cell lung cancer. Lancet. 2024;404:803–22. 39121882 10.1016/S0140-6736(24)01029-8

[30] Ebright RY, Dilly J, Shaw AT, Aguirre AJ. Response and resistance to RAS inhibition in cancer. Cancer Discov. 2025;15:1325–49. 40293709 10.1158/2159-8290.CD-25-0349 PMC12226231

[31] Jiang T, Lin C, Le S, Zhang L, Liang T, Cai L, et al. Discovery of fulzerasib (GFH925) for the treatment of KRAS G12C-mutated solid tumors. J Med Chem. 2025;68:15386–402. 40701936 10.1021/acs.jmedchem.4c03183

[32] Kruspig B, Monteverde T, Neidler S, Hock A, Kerr E, Nixon C, et al. The ERBB network facilitates KRAS-driven lung tumorigenesis. Sci Transl Med. 2018;10:1–11. 10.1126/scitranslmed.aao2565 PMC6881183 29925636

[33] Xue JY, Zhao Y, Aronowitz J, Mai TT, Vides A, Qeriqi B, et al. Rapid non-uniform adaptation to conformation-specific KRAS(G12C) inhibition. Nature. 2020;577:421–5. 31915379 10.1038/s41586-019-1884-x PMC7308074

[34] Young LC, Rodriguez-Viciana P. MRAS: a close but understudied member of the RAS family. Cold Spring Harb Perspect Med. 2018;8:1–10. 10.1101/cshperspect.a033621 PMC6280710 29311130

[35] Brannan JM, Dong W, Prudkin L, Behrens C, Lotan R, Bekele BN, et al. Expression of the receptor tyrosine kinase EphA2 is increased in smokers and predicts poor survival in non-small cell lung cancer. Clin Cancer Res. 2009;15:4423–30. 19531623 10.1158/1078-0432.CCR-09-0473

[36] Menges CW, McCance DJ. Constitutive activation of the Raf-MAPK pathway causes negative feedback inhibition of Ras-PI3K-AKT and cellular arrest through the EphA2 receptor. Oncogene. 2008;27:2934–40. 18059341 10.1038/sj.onc.1210957

[37] Paraiso KH, Das Thakur M, Fang B, Koomen JM, Fedorenko IV, John JK, et al. Ligand-independent EPHA2 signaling drives the adoption of a targeted therapy-mediated metastatic melanoma phenotype. Cancer Discov. 2015;5:264–73. 25542447 10.1158/2159-8290.CD-14-0293 PMC4355213

[38] Gouw AM, Eberlin LS, Margulis K, Sullivan DK, Toal GG, Tong L, et al. Oncogene KRAS activates fatty acid synthase, resulting in specific ERK and lipid signatures associated with lung adenocarcinoma. Proc Natl Acad Sci USA. 2017;114:4300–5. 28400509 10.1073/pnas.1617709114 PMC5410819

[39] Youngblood VM, Kim LC, Edwards DN, Hwang Y, Santapuram PR, Stirdivant SM, et al. The ephrin-A1/EPHA2 signaling axis regulates glutamine metabolism in HER2-positive breast cancer. Cancer Res. 2016;76:1825–36. 26833123 10.1158/0008-5472.CAN-15-0847 PMC4873477

[40] Huang J, Tsang WY, Fang XN, Zhang Y, Luo J, Gong LQ, et al. FASN inhibition decreases MHC-I degradation and synergizes with PD-L1 checkpoint blockade in hepatocellular carcinoma. Cancer Res. 2024;84:855–71. 38486485 10.1158/0008-5472.CAN-23-0966

[41] Cuyàs E, Pedarra S, Verdura S, Pardo MA, Espin García R, Serrano-Hervás E, et al. Fatty acid synthase (FASN) is a tumor-cell-intrinsic metabolic checkpoint restricting T-cell immunity. Cell Death Discov. 2024;10:417. 39349429 10.1038/s41420-024-02184-z PMC11442875

[42] Edwards AC, Stalnecker CA, Jean Morales A, Taylor KE, Klomp JE, Klomp JA, et al. TEAD inhibition overcomes YAP1/TAZ-driven primary and acquired resistance to KRASG12C inhibitors. Cancer Res. 2023;83:4112–29. 37934103 10.1158/0008-5472.CAN-23-2994 PMC10821578

[43] Feng J, Hu Z, Xia X, Liu X, Lian Z, Wang H, et al. Feedback activation of EGFR/wild-type RAS signaling axis limits KRAS(G12D) inhibitor efficacy in KRAS(G12D)-mutated colorectal cancer. Oncogene. 2023;42:1620–33. 37020035 10.1038/s41388-023-02676-9 PMC10181928

