# Supplementary material for: Cetuximab co-treatment with KRAS G12C inhibitors fulzerasib and sotorasib in human KRAS G12C non-small cell lung cancer cells
Source: Cell Death Discov. 2026 Mar 5;12:134. doi: 10.1038/s41420-026-02998-z (PMC13039536; doi:10.1038/s41420-026-02998-z)
Supplement: Supplementary file 1 — Revised Supplementary information-Clean version [file 41420_2026_2998_MOESM1_ESM.docx]

**SUPPLEMENTARY INFORMATION**

**Cetuximab co-treatment with KRAS G12C inhibitors fulzerasib and sotorasib in human KRAS G12C non-small cell lung cancer cells**

Daniel Olmo-González^1,2^, Mengxin Zhou^3^, Nuno G. Oliveira^4^, Fusheng Zhou^5^, Feng Yan^5^, Jèssica González^3^, Kevin València-Clua^3^, Miguel Angel Molina-Vila^6^, Jordi Bertrán-Alamillo^6^, Ana Giménez-Capitán^2,6^, Jordi Codony-Servat^3^, Rafael Rosell^1,3,7^

**
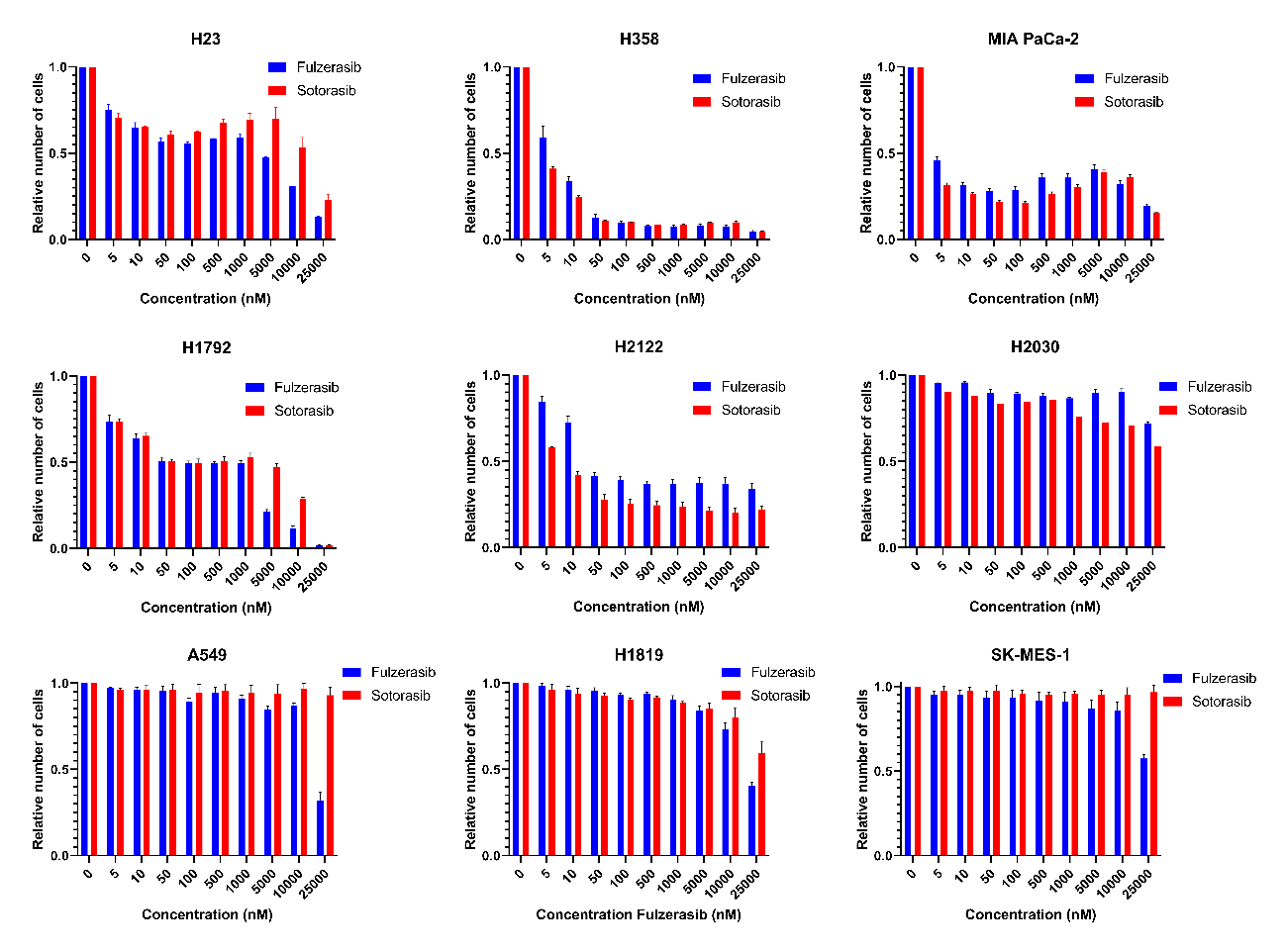
**

**Supplementary Figure 1.** Dose-response plots for fulzerasib and sotorasib in KRAS G12C-mutant, KRAS G12S, and wild-type cell lines. Values shown are means ± SD of three independent experiments. In each experiment, every drug concentration was tested in six wells (n = 6).


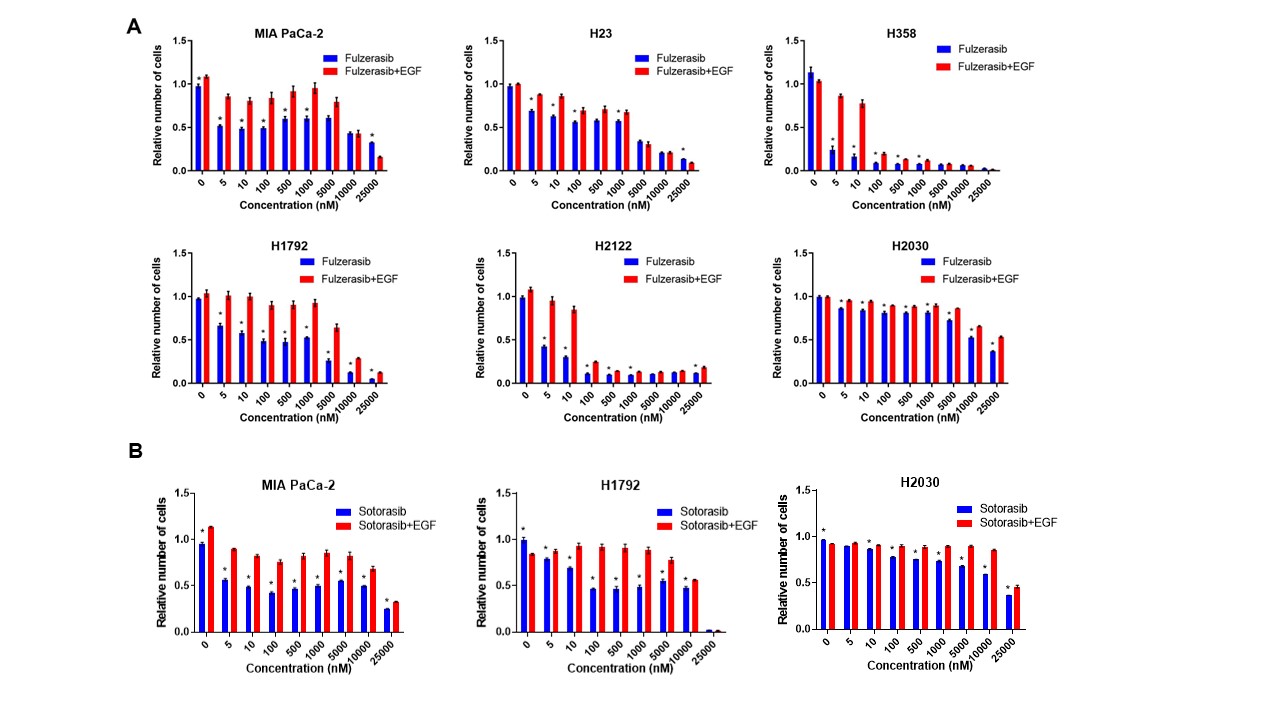


**Supplementary Figure 2**. Effects of (A) fulzerasib and (B) sotorasib in presence and absence of hEGF. Dose-response plots after 72 h in *KRAS*-mutant cell lines. Medium was RPMI+0.5% HS±10 ng/mL of hEGF. Data were pooled from three different experiments and presented as mean ± SEM. *, *p* <0.05 compared to cells with no treatment (Student’s t test).

**Supplementary Figure 3.**

**
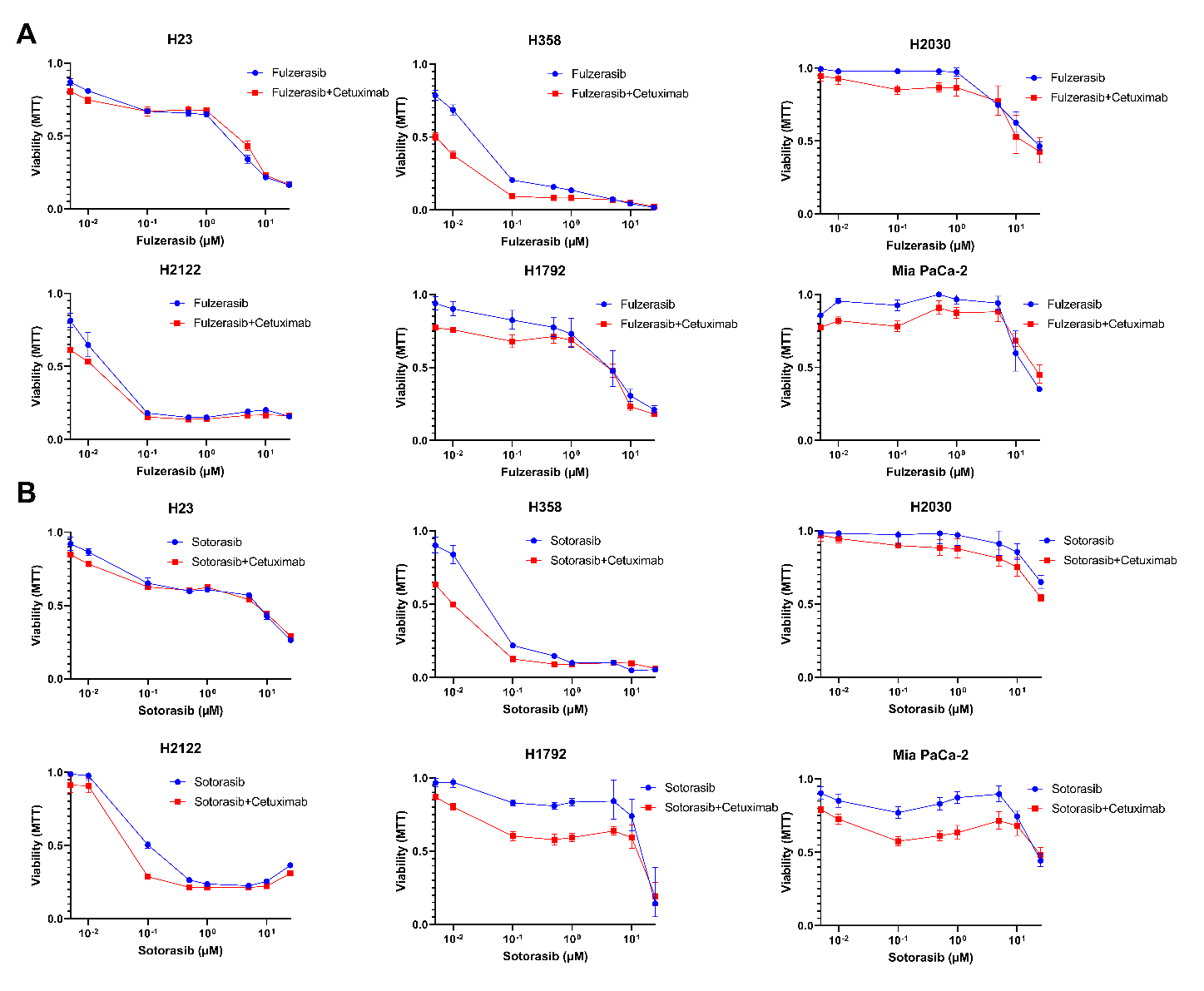
**

**Supplementary Figure 3**. Dose response effects of (A) fulzerasib and (B) sotorasib in KRAS G12C-mutant cell lines. Cell viability plots after 72 h. Medium was RPMI+0.5% HS±10 ng/mL of hEGF in KRASG12C inhibitors + cetuximab treatments. Data were pooled from three different experiments and presented as mean ± SEM.

**Supplementary Table 1:** Antibodies used in the study.

| **Antibodies** | **Source** | **Identifier** |
| --- | --- | --- |
| Phospho-EGF Receptor (Tyr1068) | Cell Signaling Technology | Cat# 3777; RRID:AB_2096270 |
| EGF Receptor | Cell Signaling Technology | Cat# 4267; RRID:AB_2246311 |
| Phospho-p44/42 MAPK (Erk1/2) (Thr202/Tyr204) | Cell Signaling Technology | Cat# 9101; RRID:AB_331646 |
| p44/42 MAPK (Erk1/2) Antibody | Cell Signaling Technology | Cat# 9102; RRID:AB_330744 |
| YAP | Cell Signaling Technology | Cat# 14074; RRID:AB_2650491 |
| EpHA2 | Cell Signaling Technology | Cat# 6997; RRID:AB_10827743 |
| EphA2 phosphorylated (S897) | Cell Signaling Technology | Cat# 6347; RRID:AB_11220420 |
| FASN | Cell Signaling Technology | Cat# 3180; RRID:AB_2100796 |
| ASS1 | Cell Signaling Technology | Cat# 70720; RRID:AB_2799790 |
| Hsp90 | Cell Signaling Technology | Cat# 4874; RRID:AB_2121214 |
| Phospho-YAP (Tyr357) | Abcam | Cat# ab62751; RRID:AB_956486 |
| MRAS | Abcam | Cat# ab176570; RRID:AB_3683691 |
| MIG6 | Santa Cruz Biotechnology | Cat# sc-137154; RRID:AB_2101524 |
| Anti-rabbit IgG, HRP-linked Antibody | Cytvia | Cat# NA934; RRID:AB_772206 |
| Anti-mouse IgG, HRP-linked Antibody | Cytvia | Cat# NA931; RRID:AB_772210 |

**Supplementary Table 2:** Cell lines used in the study

| **Cell line** | **Origin** | **Driver mutation** | **Main mutations** | **Origin** | **Growth** | **Cells/well**  **(MTT assay)** |
| --- | --- | --- | --- | --- | --- | --- |
| H23 | Lung adenocarcinoma | *KRAS* p.G12C | *ATM*; p.Gln1919Pro Heterozygous *STK11*; p.Trp332Ter Homozygous *TP53*; p.Met246Ile Homozygous  *KEAP1*; Gln193His | ATCC^1^ | Adherent | 2000 |
| H358 | Lung adenocarcinoma | *KRAS* p.G12C | *CTNNB1*; p.Thr75Ala Heterozygous Gene deletion *TP53* Homozygous | ATCC^1^ | Adherent | 2000 |
| H2122 | Lung adenocarcinoma | *KRAS* p.G12C | *TP53*; p.Gln16Leu Heterozygous  *TP53*; p.Cys176Phe Heterozygous  *KEAP1* mutated | ATCC^1^ | Suspension | 8000 |
| H1792 | Lung adenocarcinoma | *KRAS* p.G12C | *CDKN2A*; p.Trp110Ter Heterozygous *TP53*; c.672+1G>A Homozygous | ATCC^1^ | Adherent | 2000 |
| MIA PaCa-2 | Pancreatic adenocarcinoma | *KRAS* p.G12C | *TP53* p.Arg248Trp Homozygous | ATCC^1^ | Adherent | 2000 |
| H2030 | Lung adenocarcinoma | *KRAS* p.G12C | *TP53* p.Gly262Val Homozygous  *STK11* mutated | ATCC^1^ | Adherent | 2000 |
| H1819 | Lung adenocarcinoma | *TP53* | *TP53* X331_splice Homozygous  *SMARCA4* L1085Qfs*32  *KDM5C* Nonsense mutation W93* | ATCC^1^ | Adherent | 4000 |
| SK-MES-1 | Lung squamous cell carcinoma | *TP53* | *SLIT3* X210_Splice  *TP53* E298* Homozygous  *LATS1* E574* | ATCC^1^ | Adherent | 4000 |
| A549 | Lung adenocarcinoma | *KRAS* p.G12S | *SMARCA4* Q729Cfs*4  *ATR* X878_Splice  *STK11* Q37* Homozygous  *KEAP1* G333C  *CTNNA1* E865*  *PTPN14* E541*  *ARID4A* Q1087Afs*15  *TP53BP1* X362_Splice | ATCC^1^ | Adherent | 2000 |

^1^ATCC (Manassas, VA)
