# Supplementary material for: Cetuximab co-treatment with KRAS G12C inhibitors fulzerasib and sotorasib in human KRAS G12C non-small cell lung cancer cells
Source: Cell Death Discov. 2026 Mar 5;12:134. doi: 10.1038/s41420-026-02998-z (PMC13039536; doi:10.1038/s41420-026-02998-z)

**Western blot images**

All bands specified in the title appear the correspondent order.

**Figure 2**

**H23** sotorasib/fulzerasib EGFR


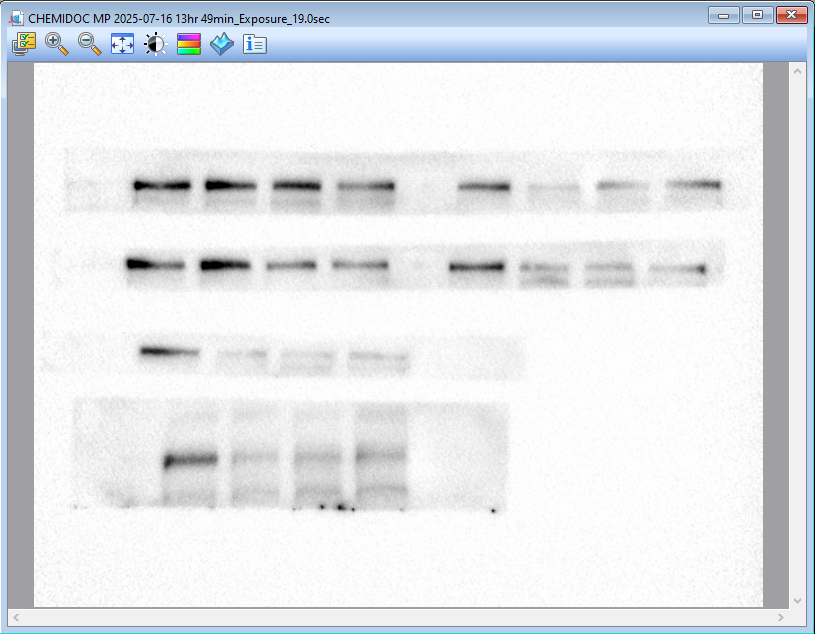


**H2030** Fulzerasib pYAP Y357 , fulzerasib +cetuximab pYAP Y357 and fulzerasib+cetuximab MRAS


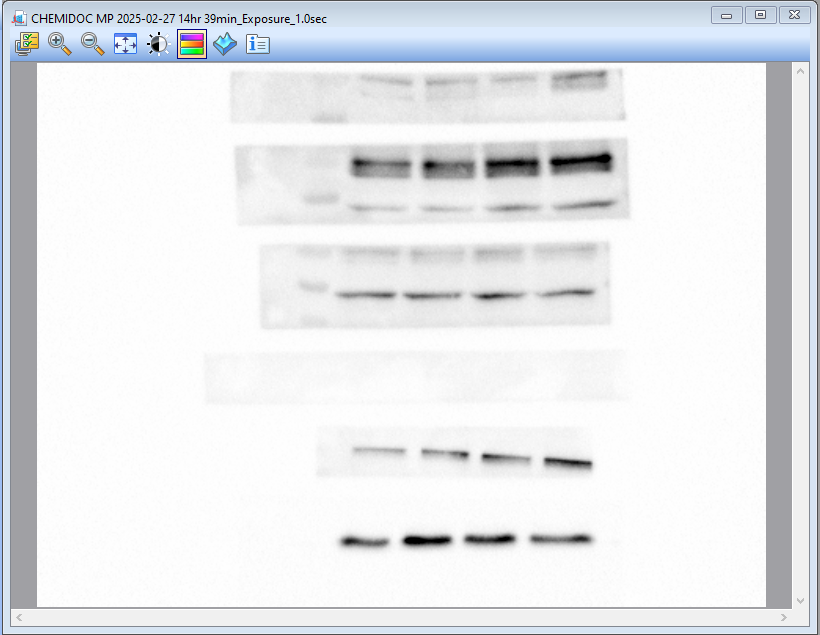


**H2030** Fulzerasib +cetuximab pERK, Fulzerasib + cetuximab pAKT, fulzerasib + cetuximab pEphA2


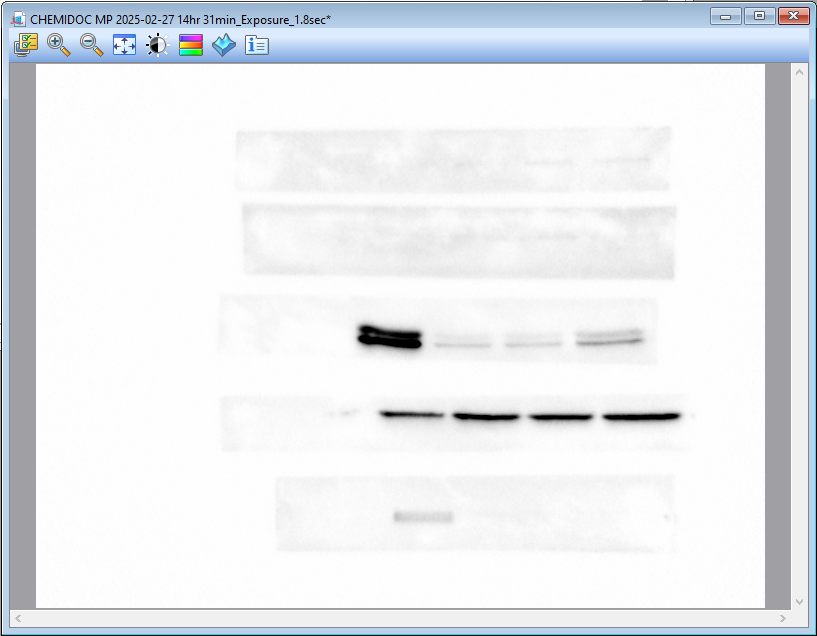


**H2030** Fulzerasib + cetuximab ERK ½, Fulzerasib + cetuximab Hsp90, Fulzerasib + cetuximab AKT, Fulzerasib AKT, Fulzerasib pEphA2


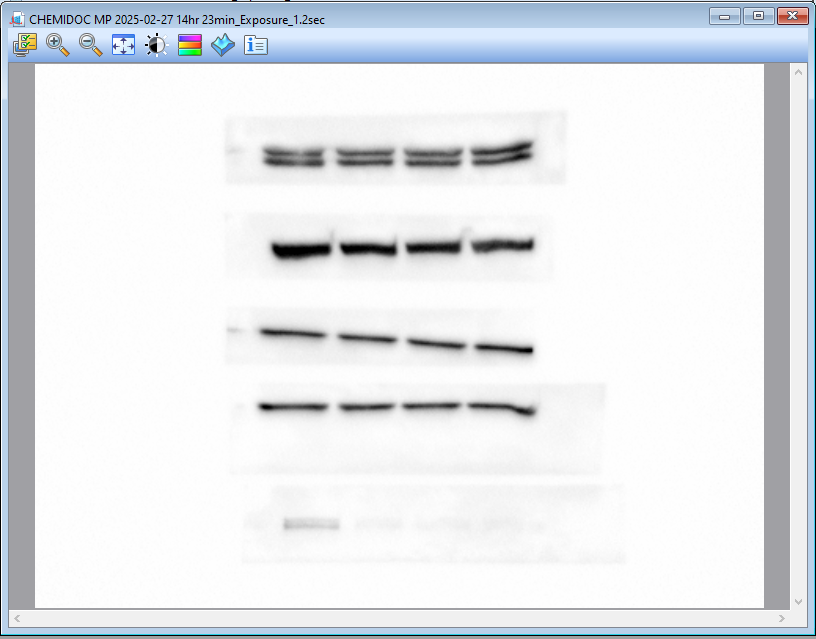


**H2030** S+C YAP, H2030 Cetuximab YAP, H2030 S+C EGFR, H2030 Sotorasib EGFR


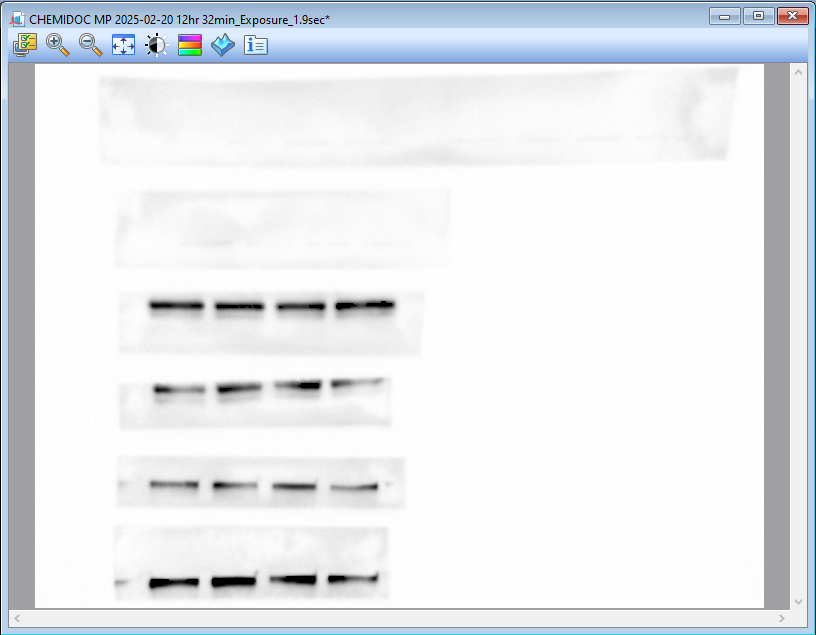


**H2030** Soto+Cet pYAP Y357, Cetuximab pYAP Y357, Soto+Cet pERK, Cetuximab pERK


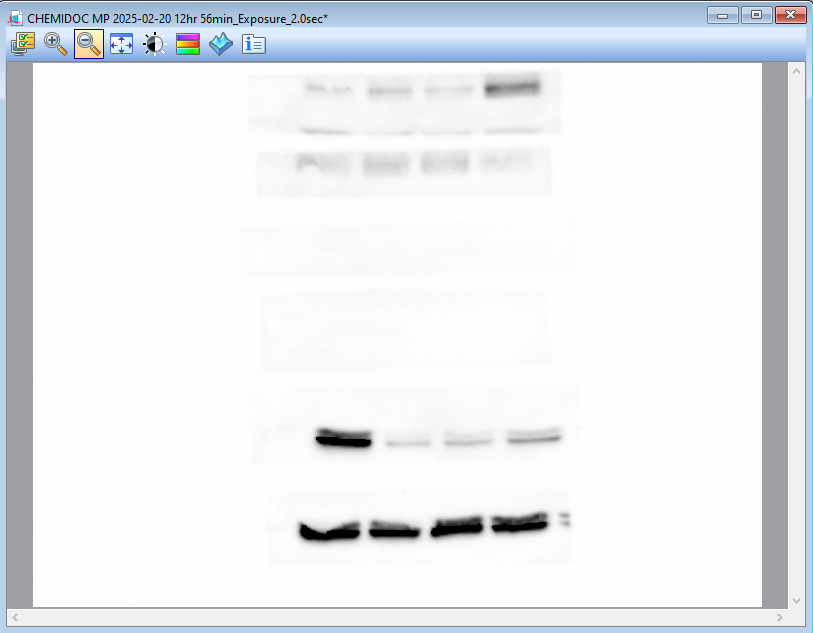


**H2030** Soto+Cet ERK, Cetuximab ERK, Soto+Cet AKT, Cetuximab AKT


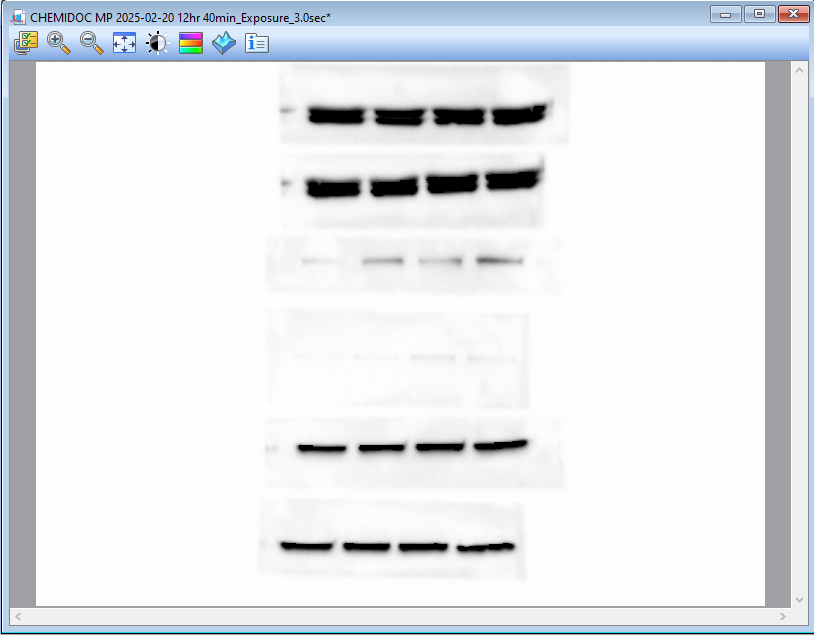


**H2030** S+C Hsp90, Soto/Cet Hsp90, Soto+Cet EPhA2, Soto ASS1, S+C ASS1


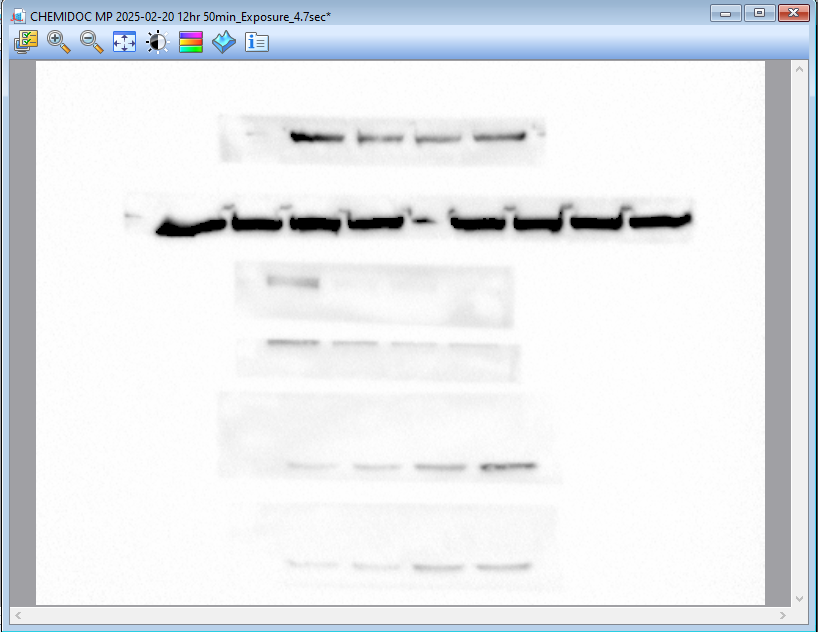


**H23** Soto+Cet pERK, **H2030** Soto pERK


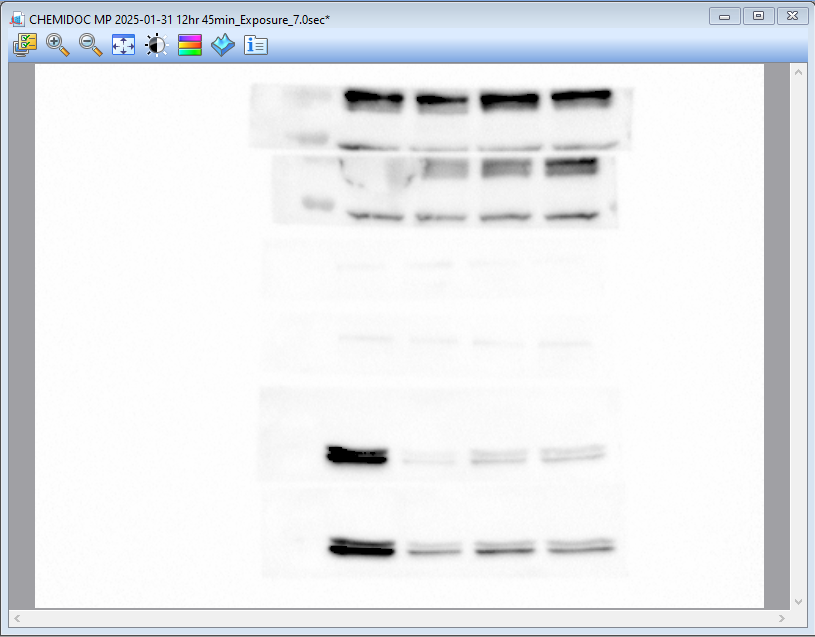


**H23** Soto+Cet pAKT, **H2030** Soto+Cet pAKT


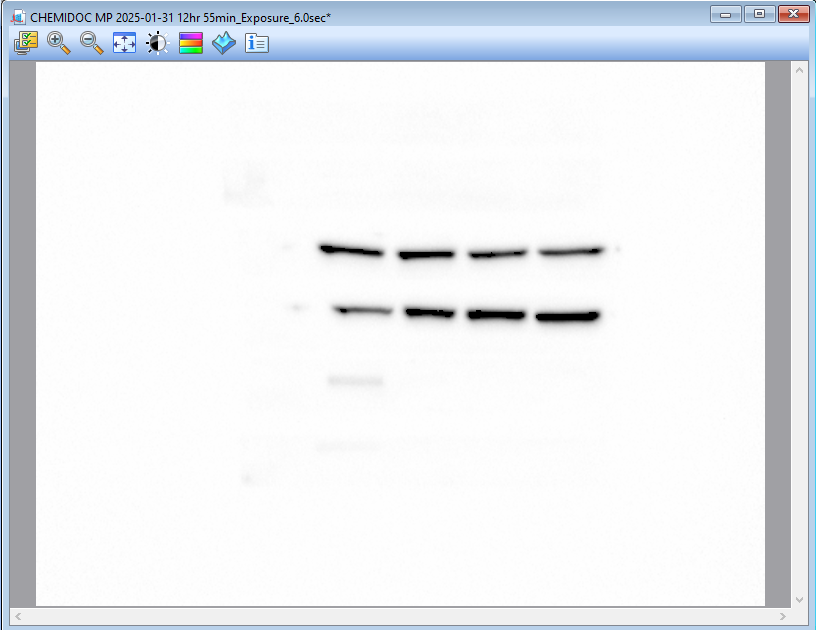


**H23** Cetuxi pYAP Y357, Cetuxi pERK 1/2


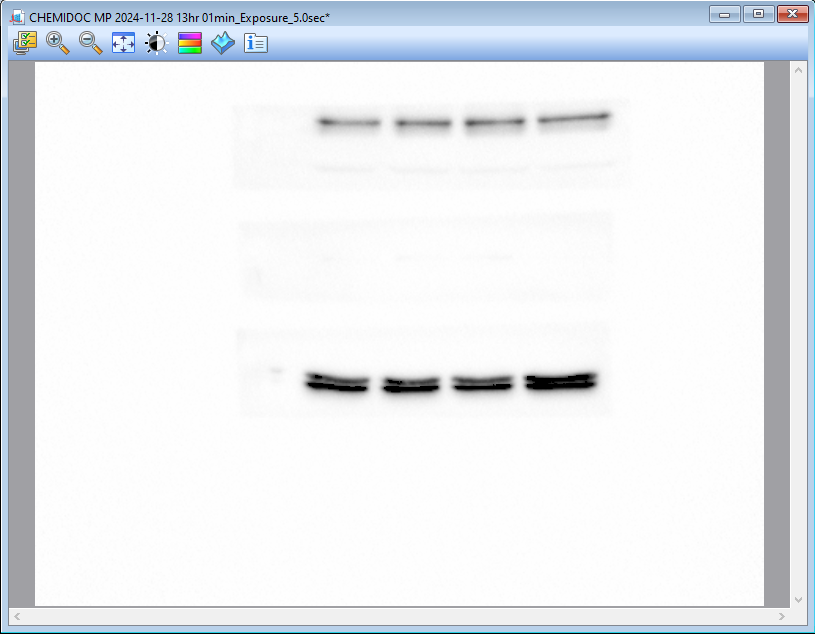


**H23** Cetuxi pAKT, Cetuximab pEphA2


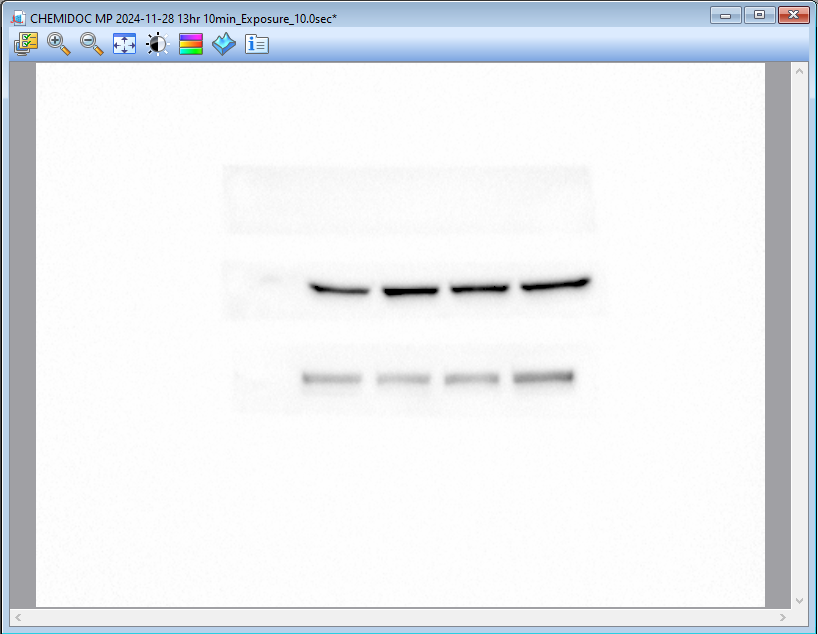


**H23** Fulzerasib FASN, Cetuximab FASN, Cetuximab EphA2


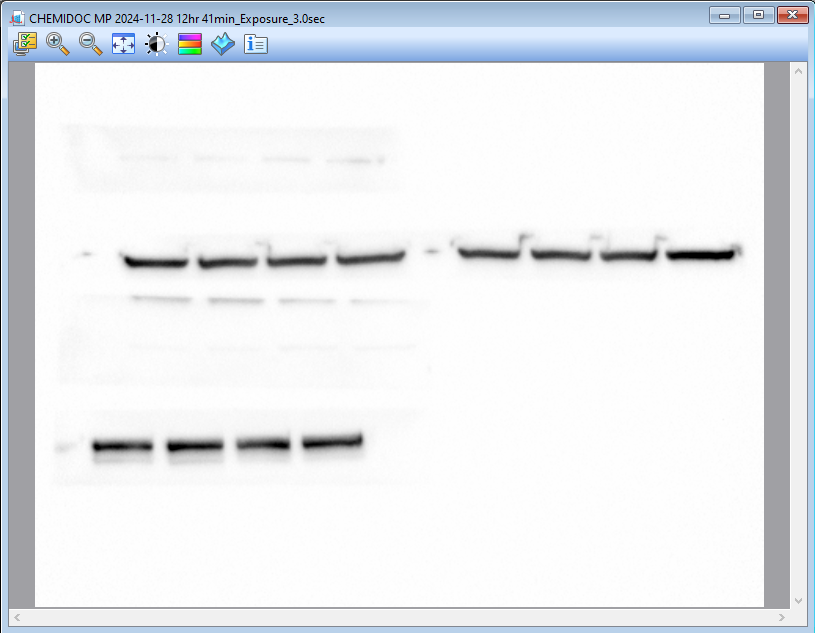


**H358** Fulzerasib pEGFR, Fulzerasib ERK, Fulzerasib YAP


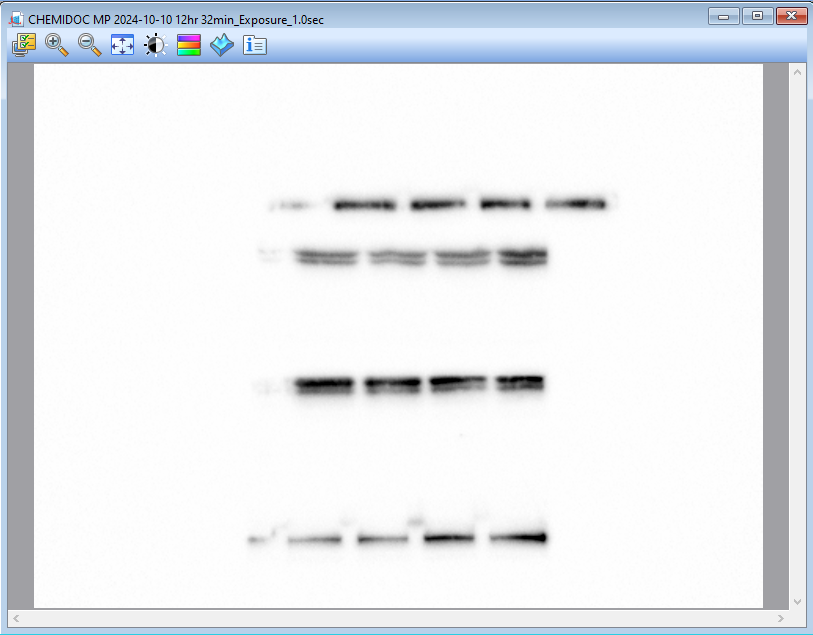


**H358** Fulzerasib pYAP Y357, pAKT S473


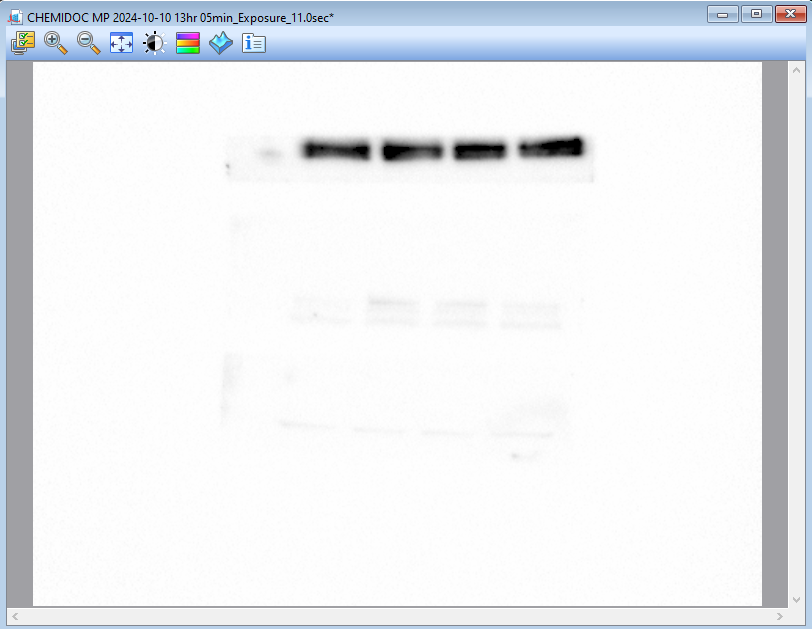


**H358** Sotorasib FASN, Sotorasib YAP, Sotorasib EGFR, Sotorasib ERK1/2


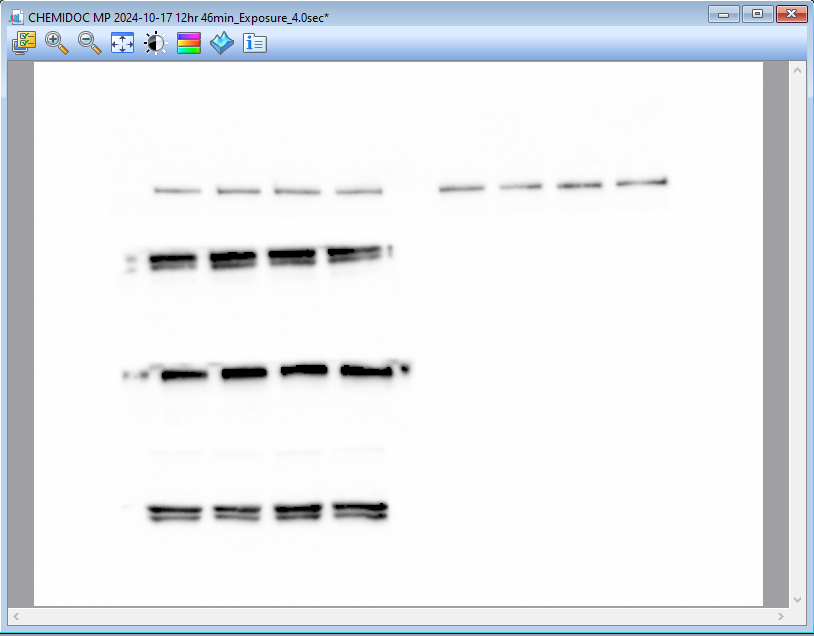


**H358** Sotorasib pAKT, Sotorasib pEphA2


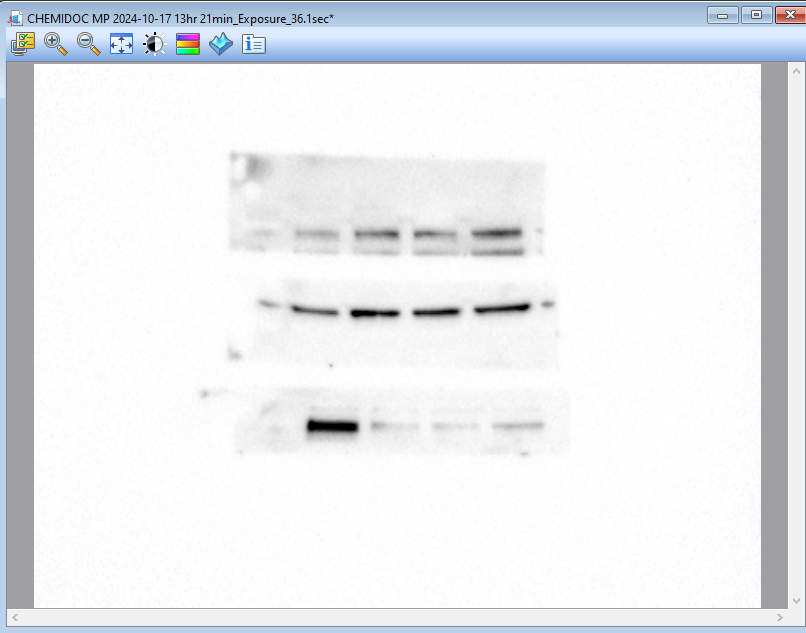


**Figure 3**

**H358** Soto/Soto+Cet MIG6, Fulzerasib/Fulzerasib+Cetuximab MIG6, Cetuximab MIG6


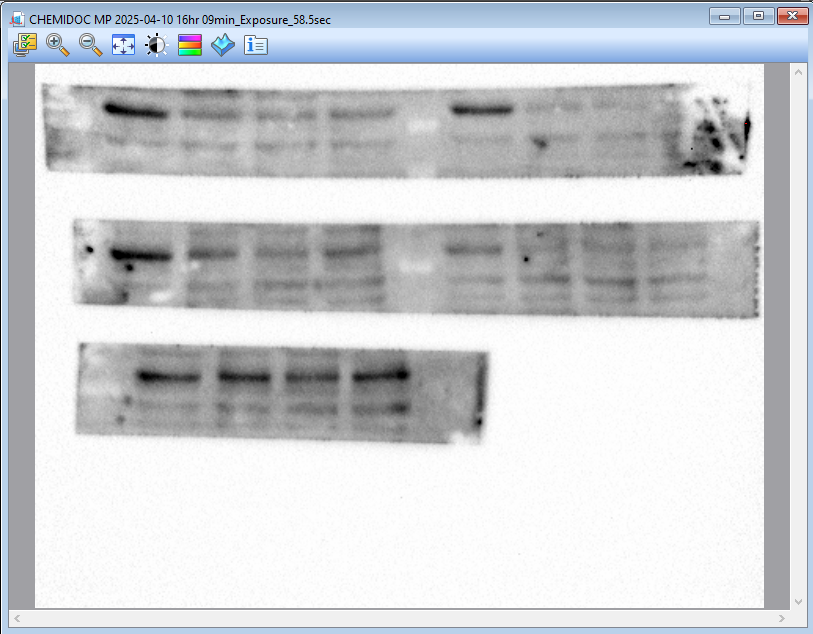


**H23** Soto/Soto+Cet MIG6, Fulzerasib/Fulzerasib+Cetuximab MIG6, Cetuximab MIG6


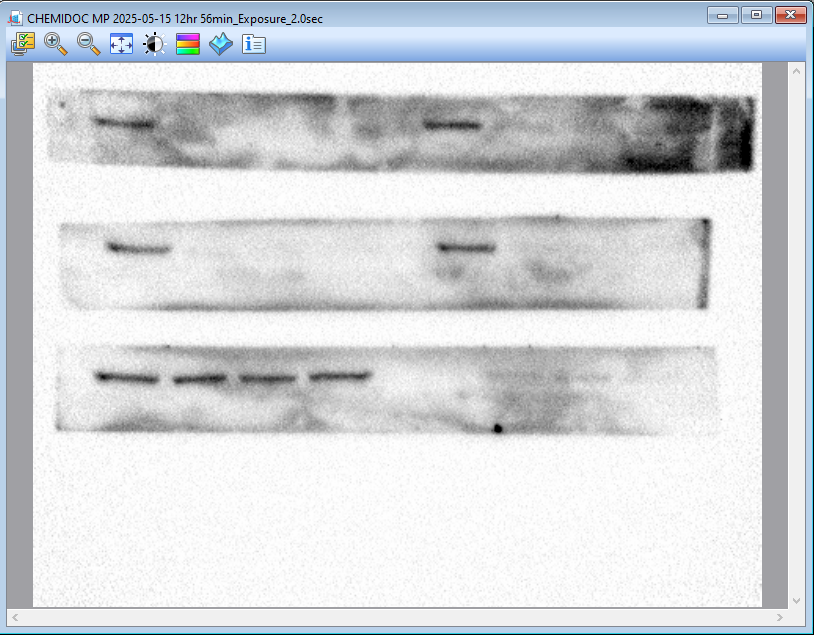


**H2030** Soto/Soto+Cet MIG6, Fulzerasib/Fulzerasib+Cetuximab MIG6, Cetuximab MIG6


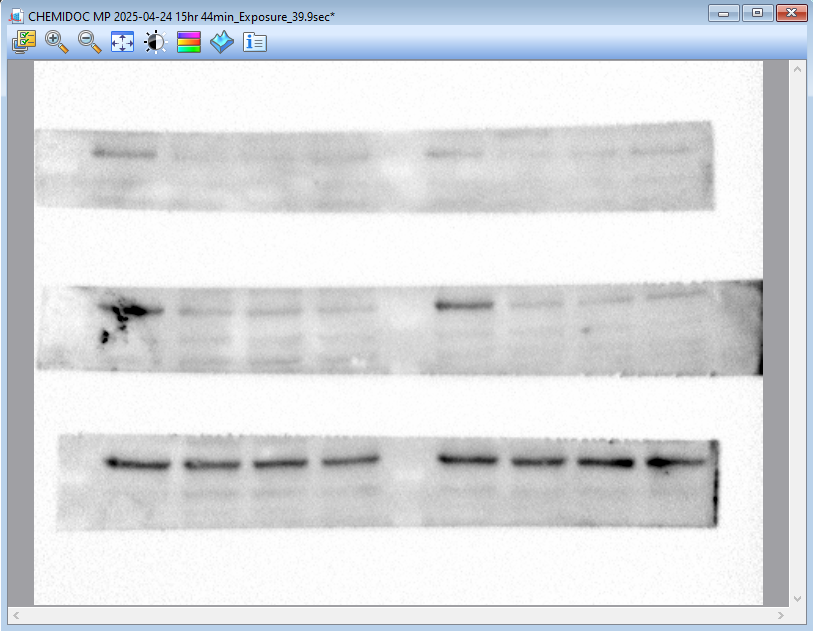


**Figure 4**

**H2030** Fulzerasib+Cetuximab ASS1


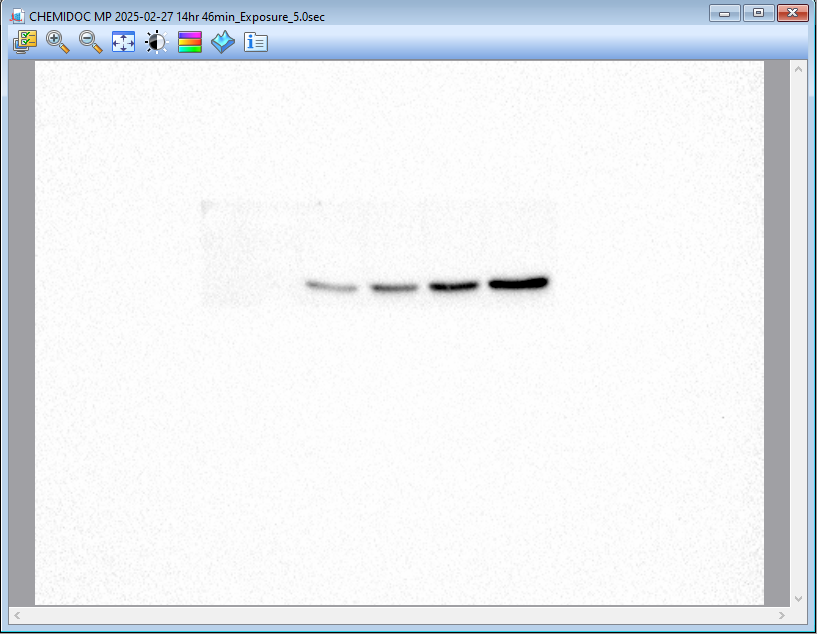


**H2030** Sotorasib + Cetuximab ASS1, Cetuximab ASS1


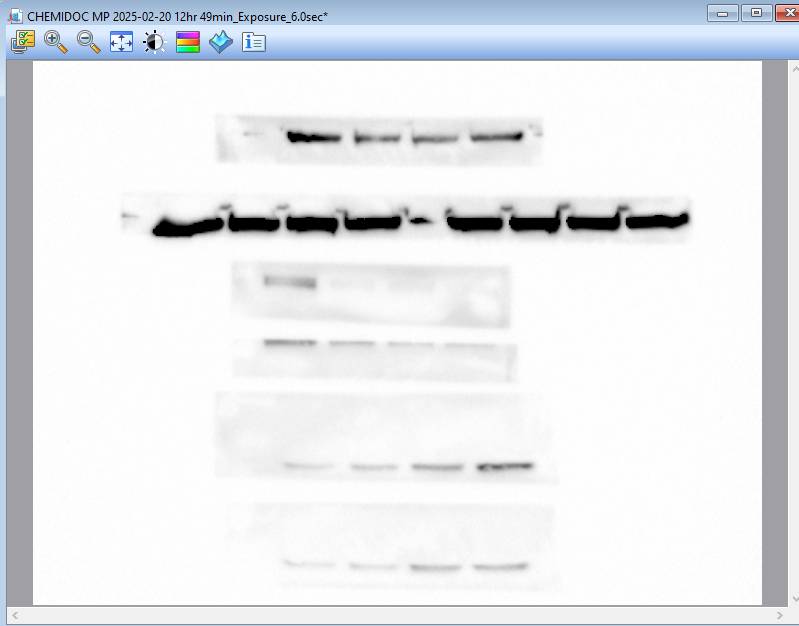


**H23** Sotaorasib + Cetuximab ASS1, **H2030** Fulzerasib


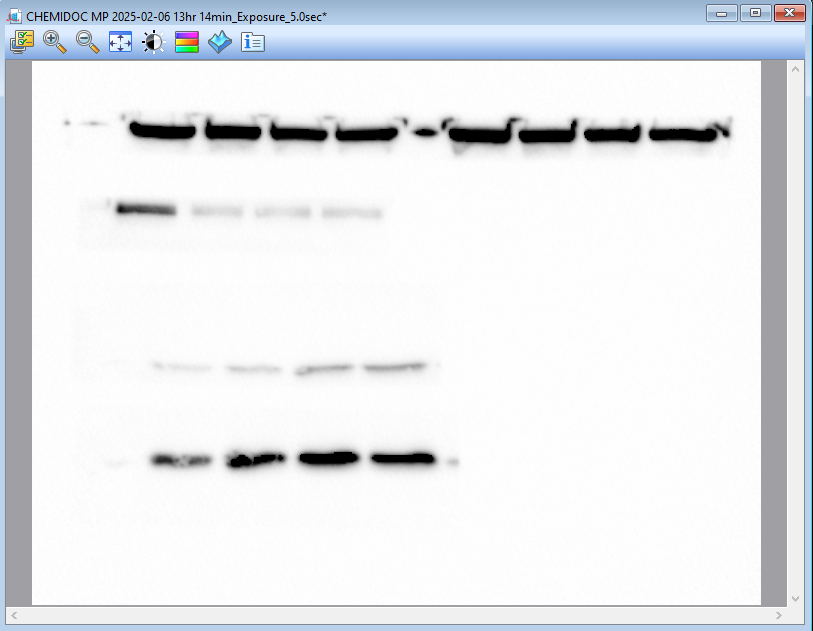


**H2030** Sotorasib


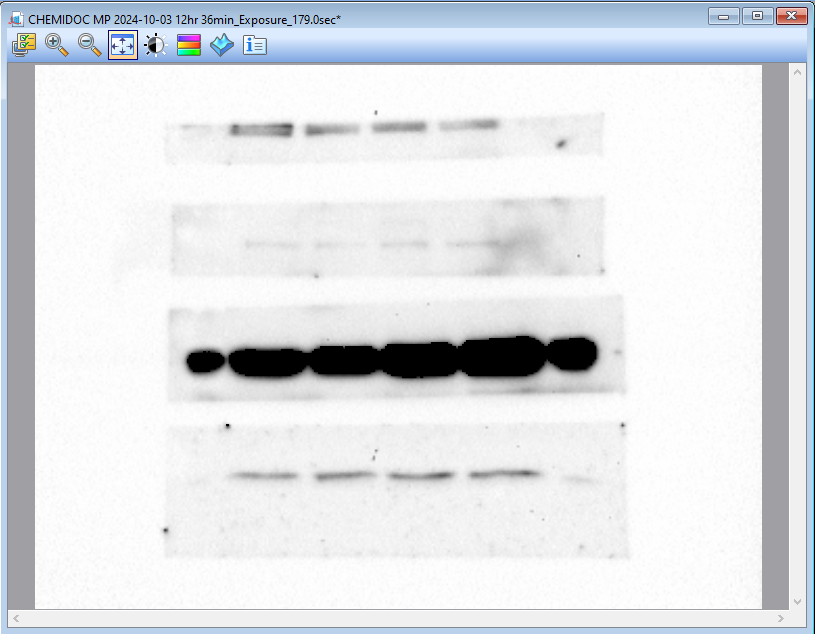


**H358** Sotorasib ASS1


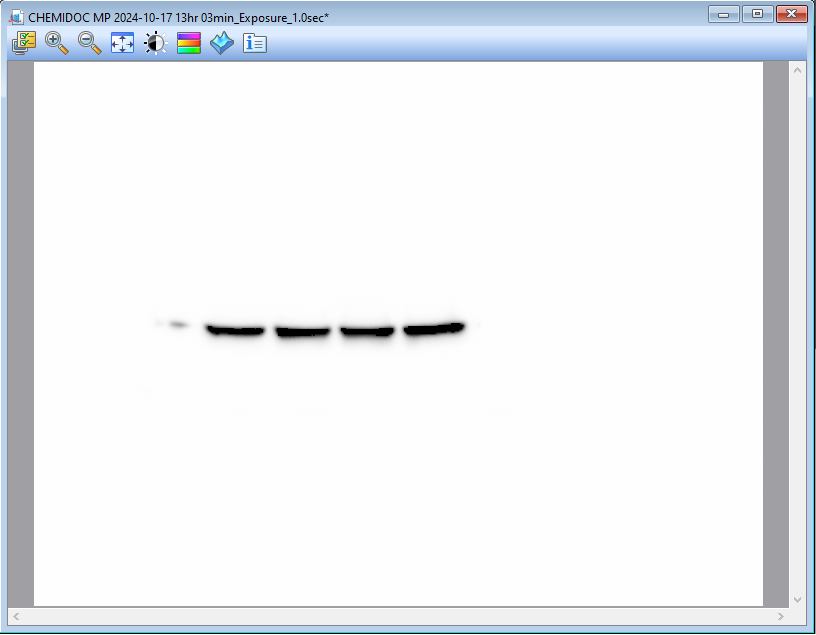

Supplement: Supplementary file 2 — Original data [file 41420_2026_2998_MOESM2_ESM.docx]
